# Integration of Parallel Opposing Memories Underlies Memory Extinction

**DOI:** 10.1016/j.cell.2018.08.021

**Published:** 2018-10-18

**Authors:** Johannes Felsenberg, Pedro F. Jacob, Thomas Walker, Oliver Barnstedt, Amelia J. Edmondson-Stait, Markus W. Pleijzier, Nils Otto, Philipp Schlegel, Nadiya Sharifi, Emmanuel Perisse, Carlas S. Smith, J. Scott Lauritzen, Marta Costa, Gregory S.X.E. Jefferis, Davi D. Bock, Scott Waddell

**Affiliations:** 1 Centre for Neural Circuits and Behaviour, The University of Oxford, Tinsley Building, Mansfield Road, Oxford OX1 3SR, UK; 2 Drosophila Connectomics, Department of Zoology, University of Cambridge, Cambridge CB2 3EJ, UK; 3 Janelia Research Campus, Howard Hughes Medical Institute, Ashburn, VA 20147, USA; 4 Division of Neurobiology, MRC Laboratory of Molecular Biology, Cambridge CB2 0QH, UK

**Keywords:** memory, extinction, dopamine, neural plasticity, parallel memory, competition, neural circuit, connectomics, *Drosophila*

## Abstract

Accurately predicting an outcome requires that animals learn supporting and conflicting evidence from sequential experience. In mammals and invertebrates, learned fear responses can be suppressed by experiencing predictive cues without punishment, a process called memory extinction. Here, we show that extinction of aversive memories in *Drosophila* requires specific dopaminergic neurons, which indicate that omission of punishment is remembered as a positive experience. Functional imaging revealed co-existence of intracellular calcium traces in different places in the mushroom body output neuron network for both the original aversive memory and a new appetitive extinction memory. Light and ultrastructural anatomy are consistent with parallel competing memories being combined within mushroom body output neurons that direct avoidance. Indeed, extinction-evoked plasticity in a pair of these neurons neutralizes the potentiated odor response imposed in the network by aversive learning. Therefore, flies track the accuracy of learned expectations by accumulating and integrating memories of conflicting events.

## Highlights

• Omission of expected punishment extinguishes learned avoidance behavior
• Lack of punishment is learned as a positive experience
• The original aversive and the new appetitive memory co-exist in different places
• Opposing memories are integrated within specific output neurons to steer behavior

## Graphical Abstract

**Figure:**
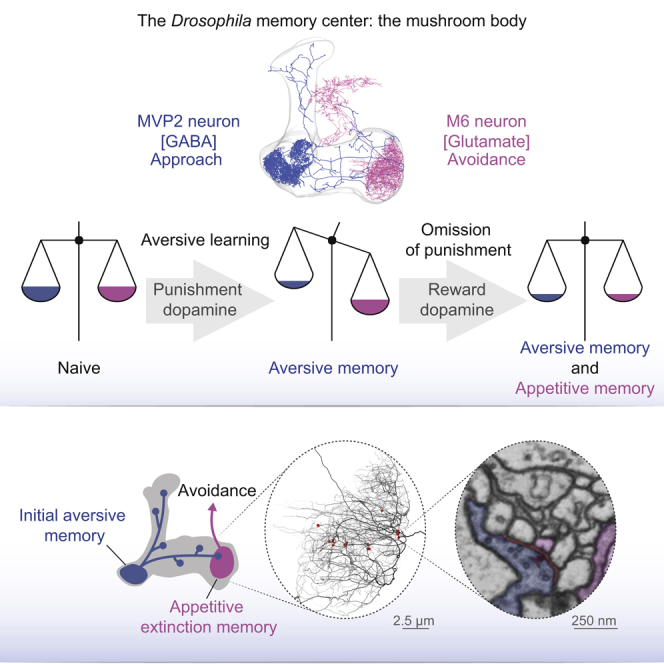

## Teaser

The omission of punishment is remembered as a rewarding experience, and this positive memory then competes against prior aversive memory to mediate the extinction of avoidance behavior.

## Introduction

Learning allows animals to predict future events. However, sometimes things do not happen when expected. Therefore, animals must also learn when expectations are not met, so that their behavior remains most appropriately directed by life experience. When humans have problems recognizing that a traumatic episode is unlikely to recur they may exhibit pathological manifestations of anxiety (Lissek and van Meurs, 2015).

A wide variety of animals can be trained to recognize that a specific sensory cue predicts pending punishment. When encountering the cue after training, they exhibit avoidance or escape behaviors, and if there is nowhere to go, they sometimes freeze. In almost all cases, learning that the cue is not such a reliable predictor, following repeated exposure without penalty, reduces the behavioral response, through a process called extinction learning. This new learning is thought to produce a parallel extinction memory, which competes with the initial aversive memory (Bouton, 2004). Many studies have indicated that extinction memory involves neural circuitry and molecular pathways different than those required for initial learning (e.g., Berman and Dudai, 2001, Bahar et al., 2003, Repa et al., 2001, Herry et al., 2010). However, it is unclear how and where extinction memories are formed and what neural network mechanisms allow extinction memories to be integrated with the initial memory, to neutralize learned behavior.

Studying extinction of olfactory memory in *Drosophila* provides an opportunity to understand the underlying neural processes at cellular resolution. Recent progress has uncovered mechanisms for the formation and expression of aversive and appetitive memories (Cognigni et al., 2018). Anatomically discrete dopaminergic neurons (DANs) provide punishment or reward teaching signals to different compartments in the mushroom body (MB) network (Claridge-Chang et al., 2009, Aso et al., 2010, Liu et al., 2012, Burke et al., 2012, Lin et al., 2014). There, dopamine release acts through dopamine receptors, especially DopR1, to drive cAMP-dependent plasticity of odor-evoked activity and ultimately presynaptic depression of specific output synapses from odor-activated Kenyon cells (KCs) (Yu et al., 2006, Kim et al., 2007, Tomchik and Davis, 2009, Qin et al., 2012, Zhang and Roman, 2013, Boto et al., 2014, Hige et al., 2015). Aversive learning depresses the relative conditioned odor drive from KCs to mushroom body output neurons (MBONs) whose activity favors approach behavior, whereas reward learning weakens connections onto MBONs directing avoidance (Séjourné et al., 2011, Owald et al., 2015, Hige et al., 2015, Perisse et al., 2016). Therefore, in simplistic terms, learning switches off certain odor-specific connections in the overall MBON network, which skews odor-driven activity toward the remaining MBON pathways either directing avoidance or approach (Aso et al., 2014b, Owald and Waddell, 2015).

An important consequence of this skewed MBON network model is that after learning, re-exposure of the trained odor drives a different configuration of the MBON network to that driven prior to and during learning. Since aversive learning switches the network into an avoidance configuration, all circuitry that lies downstream of avoidance directing MBONs should also be preferentially driven by the conditioned odor when the fly re-experiences it without punishment.

Here, we describe a neural mechanism for extinction of aversive olfactory memory in *Drosophila*. Re-experiencing a trained odor, without expected punishment drives acquisition of extinction memory. Extinction learning requires activity of a population of dopaminergic neurons, some of which are known to encode reward and are downstream of avoidance directing MBONs. Imaging odor-evoked calcium responses in the MBON network established that traces of the original aversive and new extinction memories co-exist. Anatomy and physiology demonstrate that the two memories interact within a pair of MBONs, which direct avoidance behavior. Extinction driven plasticity, measured in the dendrites of these specific MBONs (M6 or MBON-γ5β′2a), neutralizes potentiation of the trained-odor response that was imposed by aversive learning, via the release of feedforward inhibition to the M6 axons. Aversive memory performance is therefore extinguished by competition with a new memory of positive valence, formed at a different place in the MBON network.

## Results

### Aversive Memory Extinction

**Figure S1:**
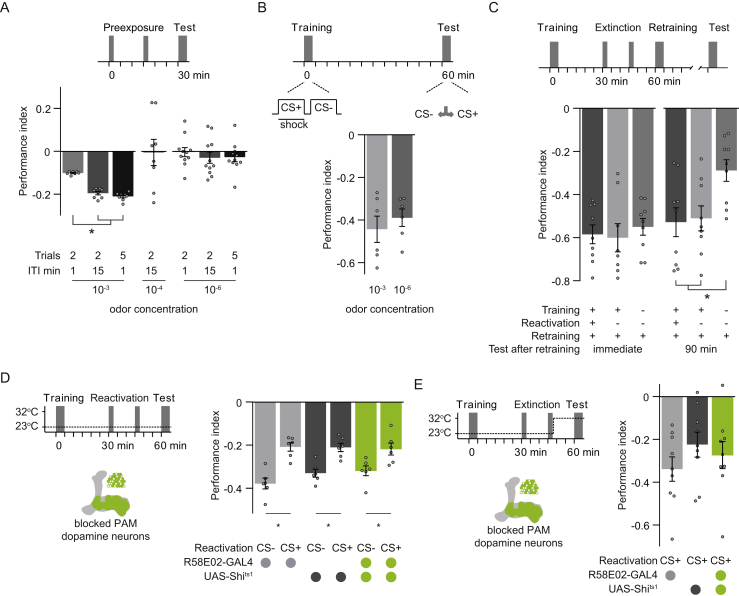
Aversive Memory Extinction Requires PAM Cluster Dopamine Neurons, Related to Figure 1 (A) Pre-exposing naive flies to high (10^−3^), but not low (10^−4^-10^−6^) concentration of odor, biases subsequent choice behavior toward avoidance of that odor in an ITI dependent manner. Low odor concentrations were therefore used in all behavioral experiments in this study. (B) Learning performance is similar with low (10^−6^) and high (10^−3^) odor concentrations. (C) Retraining after extinction reverses the reduction in learned avoidance behavior (left) and leads to more robust 90 min aversive memory (right). (D) Permissive temperature control experiment for Figure 1D. All the relevant groups show normal extinction when performance is measured 60 min after training. (E) Blocking R58E02-GAL4 dopamine neurons during the retrieval of an extinguished memory does not impair test performance. 60 min performance of R58E02-GAL4; UAS-*Shi*^ts1^ flies not statistically different from controls. Asterisks, significant difference between groups of same genotype. Data, mean ± SEM. All individual data points displayed as dots.

**Figure 1:**
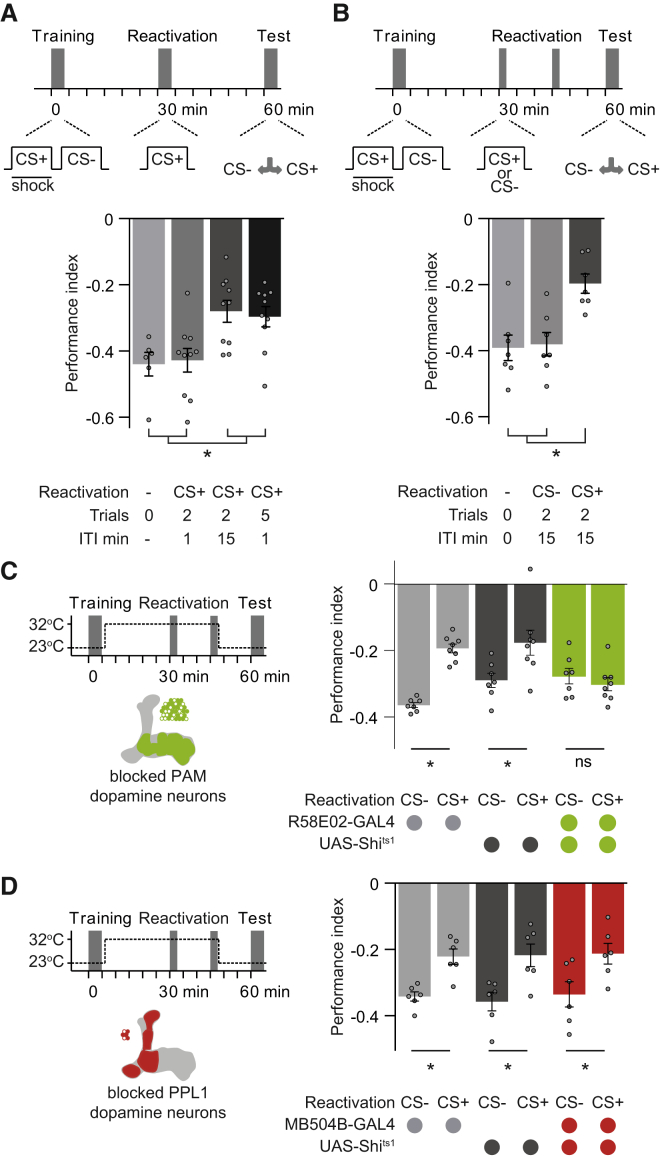
Extinction of Aversive Memory Requires PAM Dopamine Neurons (A) Top: protocol. Bottom: two (15 min ITI) or five (1 min ITI) CS+ re-exposures induces extinction. (B) Top: protocol. Bottom: CS+, but not CS−, re-exposure induces extinction. (C) Left: protocol with temperature shifting (dashed line) and R58E02-GAL4 DANs schematic. Right: blocking R58E02 neurons with UAS-*Shi*^ts1^ during CS+ re-exposure abolishes aversive memory extinction. (D) Left: protocol and MB504B-GAL4 DANs schematic. Right: blocking MB504B neurons during CS+ or CS− re-exposure does not alter extinction. Asterisks denote significant differences. Data are represented as the mean ± SEM; individual data points are displayed as dots. See also Figure S1 and Table S1.

*Drosophila* can learn to associate an odor as a predictor of forthcoming electric shock (Tully and Quinn, 1985). Following training, flies exhibit odor-specific avoidance in a T-maze. Aversive olfactory memory performance can be partially extinguished (Quinn et al., 1974, Dudai, 1977, Tully and Quinn, 1985). We first established conditions that most effectively extinguished aversive memory (Figures S1A and S1B). Flies were trained by pairing odor with 90 V shock (CS+ odor) followed by another odor alone (CS− odor). 30 min after training, flies were re-exposed to CS+ without shock to potentially extinguish memory. Finally, after a further 30 min, flies were given 2 min to choose between CS+ and CS− to test 1 hr aversive memory performance. Re-exposing flies to two spaced trials of CS+ alone (with a 15 min inter-trial interval [ITI]) or five massed CS+ trials (1 min ITI) significantly reduced learned odor avoidance, as compared to flies re-exposed to two massed CS+ trials or not re-exposed to odor (Figure 1A). Re-exposing flies twice to CS− with a 15 min ITI did not impair 1 hr aversive memory (Figure 1B). Extinction of aversive memory is therefore specifically driven by CS+ re-exposure and depends on the number of, and spacing between, CS+ extinction trials.

Learned avoidance behavior can be re-established (Figure S1C). Flies retrained 30 min after extinction show memory performance immediately after retraining that is comparable to flies whose memory has not been extinguished. Moreover, when tested 90 min later, retrained flies exhibited higher memory performance than flies only trained once. These results suggest that flies accumulate information across training, extinction, and retraining sessions (cf. Quinn et al., 1974).

### Aversive Memory Extinction Requires Dopaminergic Neurons

Extinction of appetitive memory in flies requires a small number of DANs in a cluster called PPL1 (paired posterior lateral 1), many of which can provide punishment teaching signals (Felsenberg et al., 2017). This finding suggested a plausible model for extinction where reward memory competes with an opposing aversive memory to steer behavior. We therefore tested whether aversive memory extinction required PAM (protocerebral anterior medial) cluster DANs, many of which can provide reward teaching signals. We expressed the dominant temperature sensitive dynamin UAS-*Shibire*^ts1^ (UAS-*Shi*^ts1^) transgene in PAM DANs with R58E02-GAL4 (Figure 1C). At a temperature of >29°C, Shi^ts1^ blocks membrane recycling and synaptic vesicle release, which is restored on returning to <25°C. All flies were trained at permissive 23°C, and DAN blockade restricted to the period of CS+ or CS− odor re-exposure 30 min after training, by raising the temperature to >29°C. Flies where then returned to 23°C to restore DAN function during testing. All controls subjected to this heat regimen and CS+ exposure showed normal extinction; learned avoidance was significantly reduced, compared to flies treated the same way other than being re-exposed to CS−. However, CS+ driven memory extinction was abolished when PAM DANs were blocked in R58E02-GAL4; UAS-*Shi*^ts1^ flies. Importantly, memory performance was extinguished in all groups if the entire experiment was performed at 23°C (Figure S1D). Furthermore, if PAM DANs were only blocked during testing, avoidance behavior was not different from controls (Figure S1E). We also tested whether PPL1 DANs that are necessary to acquire aversive memory were required again during extinction. Blocking these DANs specifically during odor re-exposure using MB504B-GAL4; UAS-*Shi*^ts1^ (Figure 1D) did not impair extinction. Aversive memory extinction therefore requires output specifically from PAM DANs during CS+ re-exposure.

### Avoidance-Directing MBONs Are Required for Aversive Memory Extinction and Are Functionally Connected to γ5 DANs

**Figure 2:**
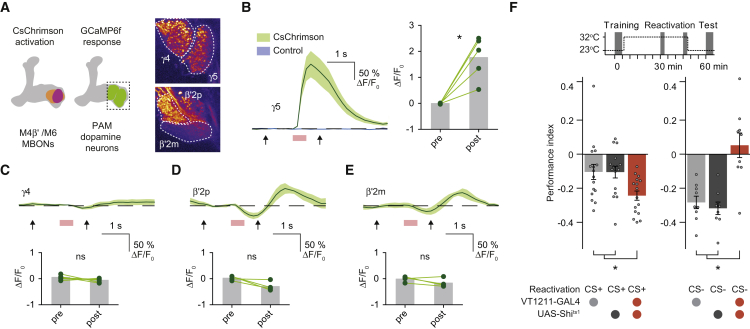
M4β′ and M6 MBONs Drive γ5 Dopamine Neurons and Memory Extinction (A) M4β′ and M6 neurons were activated with CsChrimson and GCaMP6f Ca^2+^ responses measured in presynapses of specific PAM DANs, identified by compartment innervation in the MB lobes. Insets: maximum projections of imaging planes with pseudo-colored GCaMP signals and γ4, γ5, β′2p, and β′2 m regions of interest (ROIs). Controls express GCaMP6f, but not CsChrimson. (B) M4β′/M6 activation evoked significant signals in γ5 PAM DANs. (C–E) M4β′/M6 activation did not evoke significant signals in γ4 PAM DANs (C), whereas it produced subtle inhibition and rebound in PAM β′2p (D) and PAM β′2 m (E) DANs. Arrows, time points, before and after light stimulation (red box), used for quantification. Paired measurements connected (green lines) and mean response (gray bar). (F) Top: protocol with temperature shifting. Bottom: blocking VT1211-GAL4 M4β′/M6 with UAS-*Shi*^ts1^ during CS+ re-exposure (left) reduces extinction. Blocking M4β′/M6 during CS− re-exposure abolishes odor avoidance behavior (right). Asterisk denotes significant difference. ns, no significant difference. Asterisks in (A)–(E) indicate a significant difference between pre- and post-activation responses. Data in (F) mean ± SEM. Dots represent individual data points. See also Figure S2 and Table S1.

**Figure S2:**
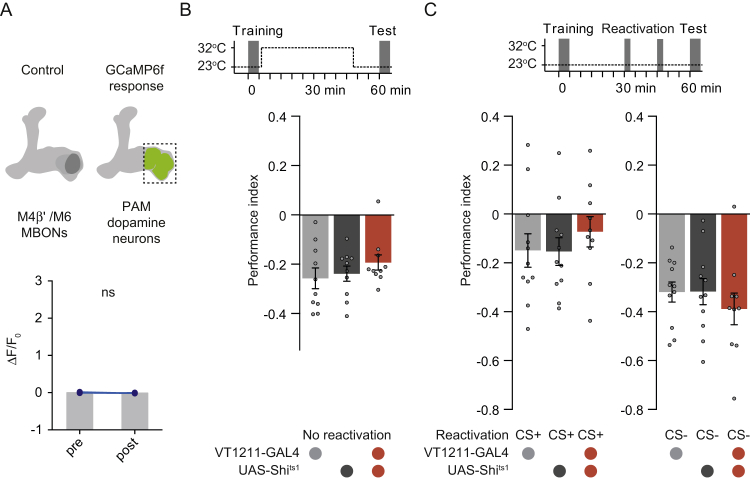
Mushroom Body Output Neurons Drive γ5 Dopamine Neurons and Memory Extinction, Related to Figure 2 (A) Control functional connectivity experiment using flies which express GCaMP6f in PAM DANs but not CsChrimson in M4β′/M6 neurons, light alone does not evoke a Ca^2+^ response in PAM DANs innervating γ5. ns, no significant difference. (B) Blocking VT1211-GAL4 labeled M4β′/M6 neurons with UAS-*Shi*^ts1^ for 45 min after aversive conditioning does not alter 60 min memory performance. (C) Permissive temperature control experiment for Figure 2F. All groups show comparable avoidance behavior after extinction (left) or after CS- re-exposure (right). Data are mean ± SEM and all individual data points are displayed (dots).

Since extinction is evoked by odor re-exposure we reasoned that the relevant PAM DANs must be driven through an olfactory neural pathway. Aversive conditioning depresses CS+ odor drive to approach-directing MBONs, such as cholinergic MBON-V2α and GABA-ergic MVP2 (MBON-γ1pedc>α/β), and potentiates CS+ responses in avoidance-promoting M4β′ and M6 neurons (MBON-β′2mp and MBON-γ5β′2a) on the horizontal MB lobe tips (Séjourné et al., 2011, Owald et al., 2015, Bouzaiane et al., 2015, Perisse et al., 2016). This plasticity therefore leaves avoidance MBONs preferentially driven by CS+ after training. In addition, presynapses from M4β′ and M6 neurons are close to dendrites of PAM DANs that innervate the same MB compartments (Aso et al., 2014a, Owald et al., 2015). We therefore tested whether PAM DANs could be driven by M4β′ and M6 activation, in explanted brains. We expressed red-light activated channelrhodopsin lexAop-CsChrimson (Klapoetke et al., 2014) with VT1211-LexA in M4β′ and M6 and light-stimulated them, while simultaneously recording fluorescence from presynaptic terminals of PAM DANs with R48B04-GAL4 driven UAS-GCaMP6f (Chen et al., 2013) (Figure 2A). Signals were assigned to discrete DANs innervating β′2, γ4, and γ5 compartments. M4β′ and M6 neuron stimulation consistently produced robust γ5 DAN excitation (Figures 2B and S2A), consistent with a recent report (Zhao et al., 2018). In contrast, γ4 DANs did not respond (Figure 2C), whereas β′2p and β′2 m DANs showed a subtle delayed inhibition and excitatory rebound (Figures 2D and 2E).

We also tested whether M4β′ and M6 MBONs are required for aversive memory extinction using VT1211-GAL4; UAS-*Shi*^ts1^ flies. Blocking M4β′ and M6 output during CS+ re-exposure, impaired memory extinction (Figure 2C). Blocking M4β′ and M6 during CS− re-exposure unexpectedly also abolished odor avoidance behavior (Figure 2C) whereas only raising the temperature without memory reactivation had no effect (Figure S2B). Extinction and avoidance behavior following CS− re-exposure were also unaltered in VT1211-GAL4; UAS-*Shi*^ts1^ flies at 23°C (Figure S2C). These data are consistent with a model in which odor re-exposure via M4β′ and M6 MBONs drives γ5 DANs to form a parallel extinction memory at the connection between CS+ odor-activated KCs and dendrites of M6 neurons.

### Extinction Memory Co-exists with the Original Aversive Memory

**Figure 3:**
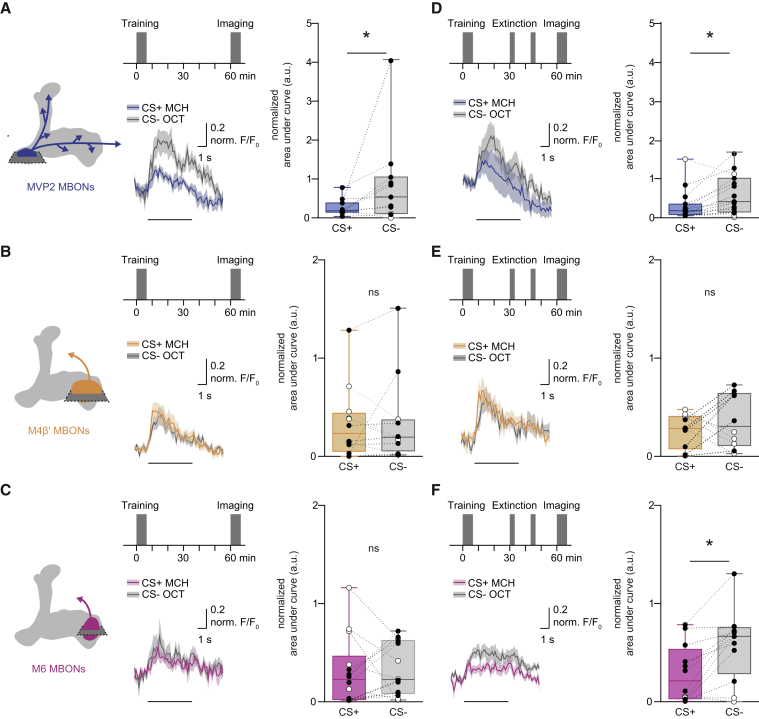
Parallel Memory Traces Form When Aversive Memory Is Extinguished (A) Imaging plane in MVP2 dendritic field, training and imaging protocol under the microscope. Aversive conditioning significantly reduces CS+ responses in MVP2. (B and C) No differences evident in M4β′ (B) or M6 (C) dendrites after aversive conditioning. (D) Extinction protocol. Training induced reduction in CS+ response in MVP2 remains after extinction. (E) Odor responses in M4β′ dendrites unchanged following extinction. (F) Extinction induces relative decrease in the CS+ response in M6 dendrites. Odor-evoked activity traces, mean (solid line) and SEM. (shadow). Black line, 5 s odor presentation. Paired measurements from individual flies shown as black (CS+ response < CS− response) or white (CS+ response > CS− response) dots. Asterisks, significant difference between averaged CS+ and the CS− responses. ns, no significant difference. See also Figures S3 and S6 and Table S1.

**Figure S3:**
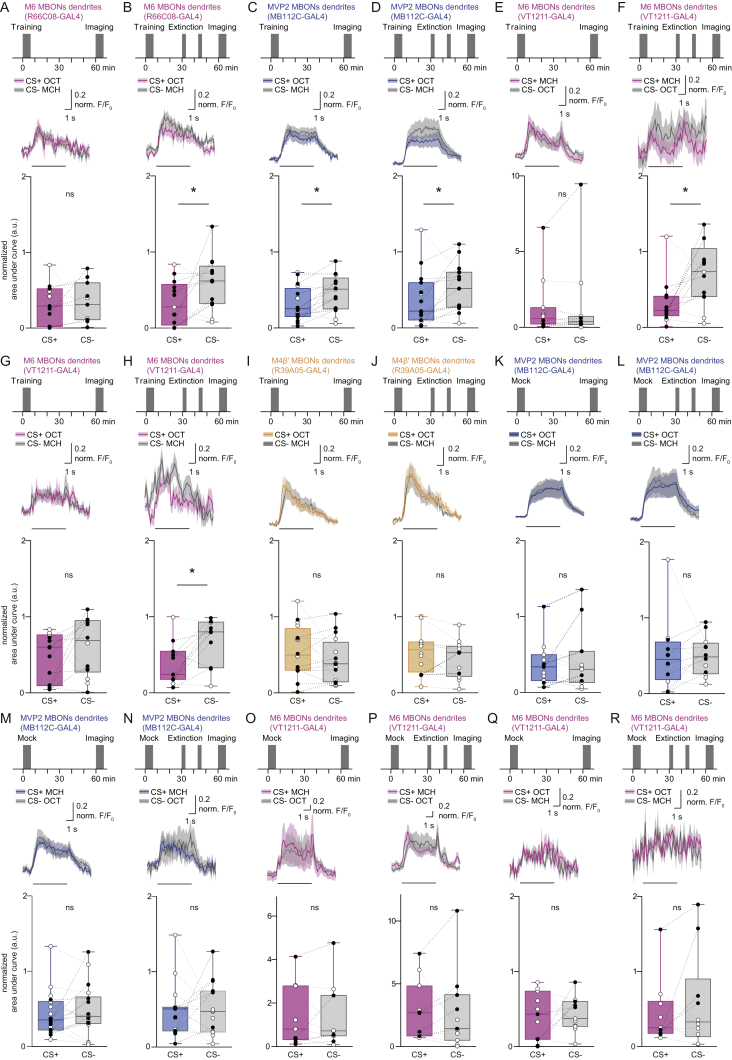
Parallel Memory Traces Form When Aversive Memory Is Extinguished, Related to Figure 3 (A and B) Complementary experiment for Figures 3C and 3F. (A) Training protocol under the microscope using 3-octanol (OCT) as CS+. Aversive conditioning does not significantly change the CS+ odor response in M6 dendrites. (B) Extinction protocol under the microscope. CS+ odor responses in M6 dendrites are significantly reduced after extinction. (C and D) Complementary experiment for Figures 3A and 3B. (C) Aversive conditioning significantly reduces CS+ odor responses in MVP2 dendrites. (D) The training induced reduction in CS+ odor (OCT) response in the MVP2 dendrites remains after extinction. (E–H) Repeating experiments in Figures 3C, 3F, S3A, and S3B with the VT1211-GAL4 driver confirms the findings: (E and G) aversive conditioning does not change odor responses in M6 dendrites. (F and H) However, aversive memory extinction leads to reduced CS+ response in M6 dendrites. (I and J) Complementary experiment for Figures 3B and 3E. (I) No differences evident in odor-evoked responses in M4β′ dendrites after aversive conditioning. (J) Odor responses measured in M4β′ dendrites are unchanged following extinction of aversive memory. (K–R) Mock conditioning, exposing flies to the same odor training regime without electric shock, does not change odor responses measured in (K and N) MVP2 or (O-R) M6 dendrites. Odor-evoked activity traces show mean (solid line) with SEM. (shadow). Black line represents 5 s odor presentation. Paired measurements from individual flies displayed as black (CS+ response < CS- response) or white (CS+ response > CS- response) dots. Asterisks, significant difference between averaged CS- and the CS+ responses.

Next, we tested whether a physiological trace of the original aversive memory survived extinction, and if so, whether a parallel extinction memory could be visualized in dendrites of M6 neurons. Flies expressing GCaMP6m in MVP2, M4β′, or M6 neurons (with MB112C-GAL4, R39A05-GAL4, or R66C08-GAL4, respectively) were prepared for odor-evoked imaging and trained under the microscope. Individuals were either left undisturbed after training or subjected to memory extinction at 30 min. CS+, CS−, and novel odor responses were measured in each fly 60 min after training and all CS+ and CS− responses were normalized to those for novel odor. Flies trained and not subjected to extinction showed the expected aversive memory trace of a relative depression of CS+ responses in MVP2 dendrites (Figure 3A). No trace of aversive memory was evident in these flies in the dendrites of M4β′ (Figure 3B) or M6 neurons (Figure 3C); CS+ and CS− responses were equivalent. However, following extinction, flies maintained a significant decrease in the relative response to CS+ in MVP2 neurons (Figure 3D). In addition, although no change was evident in odor responses measured in dendrites of M4β′, a significantly decreased response to CS+ emerged in M6 dendrites after extinction (Figures 3E and 3F). Importantly, training and extinction-induced changes in odor responsiveness of MVP2 and M6 neurons were also present when the reciprocal odor was used as CS+ (Figures S3A–S3D), and when GCaMP6f was expressed in M6 using VT1211-GAL4 (Figures S3E–S3H). No changes were observed in M4β′ dendrites in experiments using reciprocal odor as CS+ (Figure S3I and S3J). Lastly, flies subjected to mock training did not display the learning or extinction-induced differences between odor responses (Figures S2K–S2R). These data suggest that an aversive memory trace remains after extinction and that a parallel memory of opposing valence is formed elsewhere in the MBON network. Finding a relative depression of CS+ responses in M6 MBONs after extinction suggests that aversive memory extinction resembles appetitive olfactory learning reinforced with a sugar reward (Owald et al., 2015).

### MVP2 Makes Different Inhibitory Synapses with M4β′ and M6 Neurons

An MVP2-M4β′/M6 neuron connection could allow aversive and extinction memories to interact. Artificially triggering MVP2 neurons inhibited odor-responses in M4β′/M6 neurons, and light microscopy suggested MVP2 neurons might directly synapse onto primary neurites of M4β′ and M6 neurons (Perisse et al., 2016). In addition, aversive learning lead to relative potentiation of CS+ responses in M4β′ and M6 axons (Owald et al., 2015), which could result from release of CS+ specific feedforward inhibition through MVP2 neurons (Perisse et al., 2016). Lastly, since odor-responses measured in M4β′ and M6 dendrites did not show obvious change after aversive learning (Figures 3B and 3C), we wondered whether placement of MVP2 inhibitory input to M4β′ and M6 neurons would be important.

**Figure 4:**
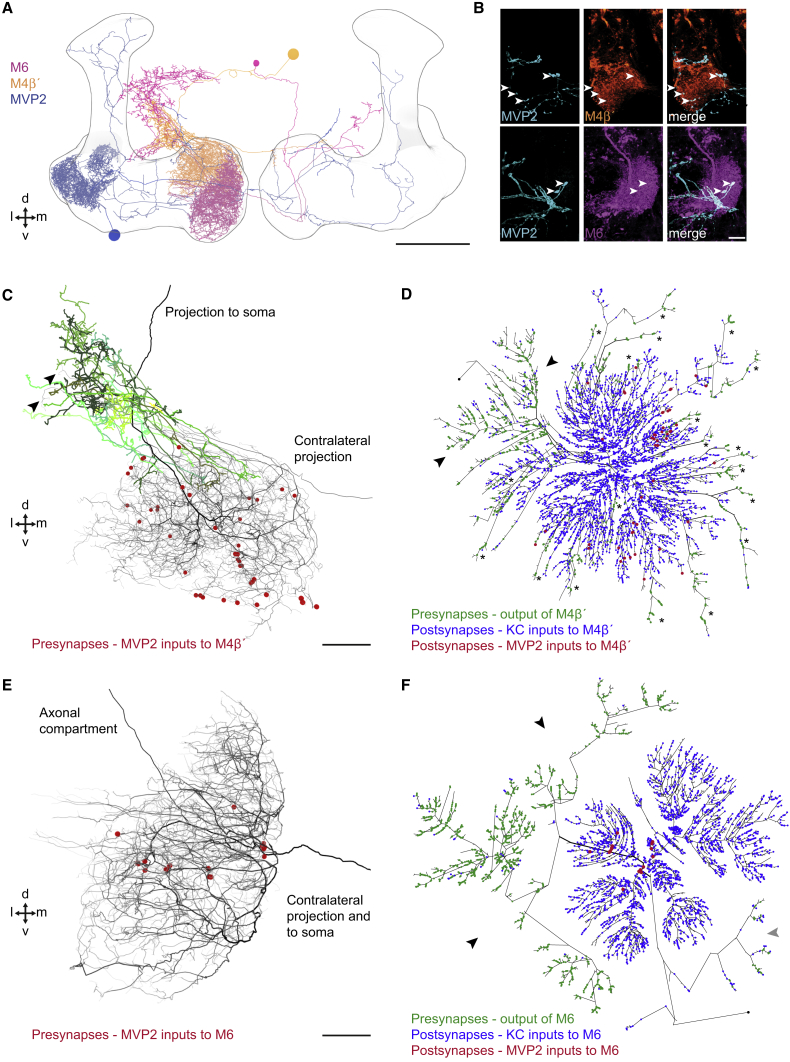
MVP2 Neurons Connect in Different Ways to M4β′ and M6 Neurons (A) 3D view of right-brain hemisphere MVP2, M4β′, and M6 neurons from EM tracing. MVP2, M4β′, and M6 neurons have ipsi- and contralateral processes in MB lobes (black outline). Scale bar, ∼20 μm. Dorsal, ventral, medial, and lateral directions indicated. See also Video S1. (B) Confocal projections of MVP2 (cyan), M4β′ (orange), and M6 (magenta), where they intersect in MB lobes. Top row: MVP2 processes intermingle with M4β′ peripheral dendrites in β′2 (white arrows). Bottom row: large diameter MVP2 axon branches (cyan) overlay M6 dendrites in γ5 (white arrows). Scale bar, 10 μm. See also Videos S2 and S3. (C) EM tracing of MVP2 inputs to M4β′ dendrites. MVP2 presynapses (red dots) opposing M4β′ postsynapses. M4β′ axonlets, presynaptic processes extending from dendritic fields are marked (green shades). Two dendritic branches project into crepine (arrows). Scale bar, ∼5 μm. (D) Dendrogram of M4β′ neuron showing postsynapses opposing MVP2 inputs (red dots), KC inputs (blue), presynaptic output (green). Neurite length not preserved. Main axon branches, arrows. Asterisks, axonlets. (E) EM tracing of MVP2 input to M6 dendrites. Annotated as in C. MVP2 inputs (red) often cluster along M6 major neurites. Scale bar, ∼2.5 μm. (F) Dendrogram of M6. MVP2 input (red), KC input (blue), presynaptic output (green). Two primary axon branches (black arrows). Contralateral axon (gray arrow). See also Figure S4.

We revisited MVP2-M4β′/M6 neuron connectivity (Figure 4A; Video S1) using light and electron microscope data. We first imaged brains from VT1211-LexA/UAS-mCD8::GFP;MB112C/lexAop-rCD2::RFP flies using an Airyscan equipped confocal microscope. Analysis of 3D reconstructed data revealed different patterns of MVP2 innervation in the vicinity of M4β′ and M6 dendritic fields. Whereas MVP2 terminals were visible around the periphery of M4β′ dendrites (Figure 4B; Video S2), MVP2 processes in M6 dendrites were larger in diameter and appeared to follow M6 neurites (Figure 4B; Video S3).

Video S1. EM Traced and Reconstructed MVP2, M4β′, and M6 Neurons, Related to Figure 4A Neurons traced from FAFB are represented within volume of the mushroom body lobe neuropil (black outline). Consistent with Aso et al. (2014a), M6 (magenta) dendrites mostly occupy the γ5 compartment with a few additional processes entering the β′2a subcompartment. Axons project into the SMP. M4β′ (orange) dendrites innervate the β′2mp subcompartments. The primary axonal field is in the crepine and an additional axonal process projects to the contralateral MB. MVP2 (blue) dendrites occupy the γ1 compartment and the αβ domain of the base of the peduncle. MVP2 neurites project throughout the vertical and horizontal MB lobes, into the crepine and also send a contralateral projection to the other MB. On the ipsilateral side MVP2 projects into the β′2 and γ5 compartments where it makes connections with the M4β′ and M6 neurons, respectively.

Video S2. 3D Maximum-Intensity Projection View of Airyscan Showing MVP2 Processes and M4β′ Dendrite, Related to Figure 4B Intersection between the MVP2 neurons (white) and dendritic field of an M4β′ neuron (red). The MVP2 processes intermingle with M4β′ peripheral dendrites. MVP2 also makes contacts onto currently unidentified postsynaptic partners (bottom right). The projected volume is 51.5x41.5x16.5μm and rolls to a maximum of 45 degrees around the X- and y axis.

Video S3. 3D Maximum-Intensity Projection View of Airyscan Showing MVP2 Processes and M6 Dendrite, Related to Figure 4B Intersection between an MVP2 neuron (white) and the dendrites of an M6 neuron (red). Large diameter MVP2 processes project within the M6 dendritic region. MVP2 also makes contacts onto currently unidentified postsynaptic partners (bottom and top left). The projected volume is 51.5x41.5x22.5 μm and rolls to a maximum of 45 degrees around the X- and y axis.

**Figure S4:**
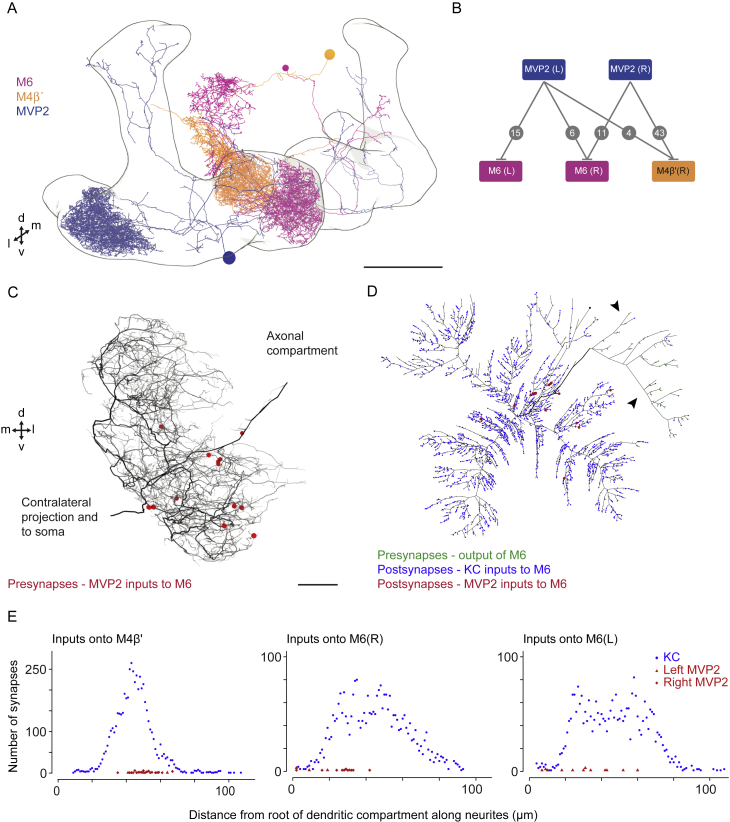
MVP2 Neurons Connect in Different Ways to M4β′ and M6 Neurons, Related to Figure 4 (A) Alternative 3D view of the projections of the fly’s right brain hemisphere MVP2, M4β′ and M6 neurons from EM tracing. MVP2 processes in the horizontal MB lobe, innervate β′2 and γ5 compartments, occupied by dendrites of M4β′ and M6 neurons, respectively. Scale ∼20μm. Dorsal, ventral, medial and lateral directions indicated. (B) Analysis of ultrastructural connectivity between MVP2 neurons from right and left hemispheres with the right hand M4β′ and right and left M6 neurons. Numbers of synapses between respective neurons indicated on connections between boxes. (C) Analysis of dendritic field of left M6(L) neuron confirms that MVP2 inputs (red dots) localize near the root of dendrites. Scale ∼2.5 μm. (D) Dendrogram of placement of MVP2 inputs to M6(L). MVP2 input (red), likely-KC input (blue), presynaptic output (green). Two primary axon branches indicated (black arrows). (E) Quantification of localization of MVP2 input to M4β′ and M6(R) and M6(L) neurons with respect to their distance to root of the dendritic field. Non-MVP2 inputs, assumed to be mostly KCs, onto M4β′, M6(R) and M6(L) neurons have a Gaussian or bimodal Gaussian distribution, spread over the dendritic field. MVP2 inputs to M4β′ are more distally localized than MVP2 inputs to M6(R) and M6(L) neurons.

Next, we took advantage of a recently acquired electron microscope (EM) volume of a full adult female fly brain to study MVP2-M4β′ and MVP2-M6 neuron connectivity at higher resolution (Zheng et al., 2018). We extensively traced complete skeletons of the fly’s right side MVP2, M4β′, and M6 neurons and examined whether they were directly connected (Figures 4A, 4C–4F, and S4A–S4D). MVP2 forms many synaptic bouton-type connections within distal sections of the M4β′ dendritic field (Figures 4C, 4D, and S4E). In contrast MVP2 forms fewer *en passant* synapses along the primary neurite and more proximal sections of M6 at the root of the dendritic tree (Figures 4E, 4F, and S4A–S4E). This placement of MVP2 synapses on the M6 neuron could shunt activity from entire dendritic branches and/or the complete dendritic tree.

**Figure 5:**
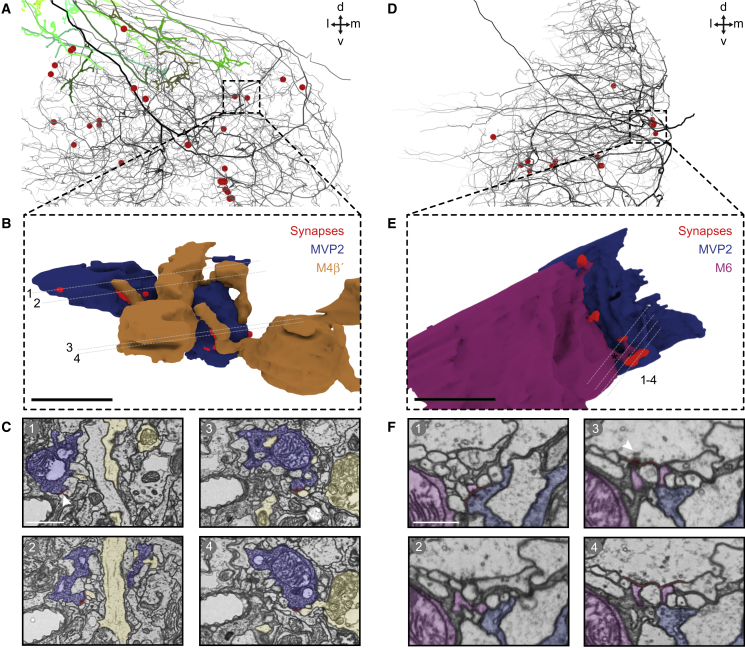
Ultrastructure of MVP2 Synapses onto M4β′ and M6 Neurons (A) 3D view of EM tracing of MVP2 presynapses within M4β′ dendritic field (same as Figure 4C). Most MVP2 presynapses are on bouton-like structures. (B) Inset, area modeled in 3D reconstruction of MVP2 bouton (blue) and corresponding presynapses (red) making inputs to different M4β′ dendrites (orange). Scale bar, 1 μm. Gray lines (1–4), locations of EM data shown in (C). (C) 1, EM image of MVP2 synapse onto unrelated neuron (white arrow). 2–4, MVP2 to M4β′ synapses. This MVP2 bouton also inputs to other neurons. Synaptic cleft (red). Scale bar, 1 μm. (D) 3D view of MVP2 presynapses within M6 dendrite (same as Figure 4E). MVP2 makes *en passant* synapses onto spines in M6 dendritic field. Scale bar, 1 μm. (E) Inset, area modeled in 3D reconstruction of *en passant* MVP2 (blue) presynapses (red) onto spine-like twigs (asterisk) of M6 (magenta). Gray lines (1–4), locations of EM sections in (F). (F) 1, presynapse (red) from MVP2 (R) (blue) onto postsynapse of M6 spine-like twig (magenta). 2, section between synapses. 3, white arrow MVP2(L) presynaptic site (red). 4, synaptic cleft (red) of same presynapse extending into two postsynaptic sites (inputs) onto M6 spine-like twig (magenta). Scale bar, 500 nm. See also Videos S4 (MVP2-M4β′) and S5 (MVP2-M6).

We also visualized morphology of individual synaptic connections with volume reconstructions of profiles in sections of the M4β′ and M6 dendritic fields, where they contact MVP2 neurons (Figure 5). A representative MVP2 bouton was found forming synapses with multiple M4β′ dendritic protrusions (Figures 5A–5C; Video S4). In contrast MVP2 makes multiple synaptic connections onto spine-like twigs protruding from the large diameter neurite of M6 (Figures 5D–5F; Video S5). Therefore, both placement and morphology of MVP2 connections to M4β′ and M6 neurons are unique.

Video S4. 3D Animation of the Ultrastructure of an MVP2 Presynaptic Bouton and Postsynaptic M4β′ Dendrites, Related to Figure 5B An MVP2 bouton (blue) and M4β′ dendrites (orange) make synaptic connections (red). MVP2 also makes contacts onto currently unidentified postsynaptic partners. The entire structure visible in the first frame has a diameter of 4μm.

Video S5. 3D Animation of MVP2 “En Passant” Synapses onto M6 Postsynaptic Spine-like Twigs, Related to Figure 5E A section of the main neurite of the M6 neuron (magenta) that is close to the root of the dendritic field can be seen to receive clustered synaptic input (red) on spine-like twigs. The MVP2 (R) neuron (blue) provides input to one of these spines. Another large synapse onto two branches of the same spine is from MVP2 (L); the synapses are indicated, but the MVP2(L) neuron is not shown. The whole structure visible in the first frame has a diameter of 3.5μm.

### Aversive and Opposing Extinction Memories Are Integrated in M6 Neurons

Since aversive memory extinction caused a decrease in conditioned odor drive to M6 dendrites, we tested whether activity in M4β′ and M6 neurites might reveal integration between the physiological effects of the original aversive memory and the new extinction memory.

**Figure 6:**
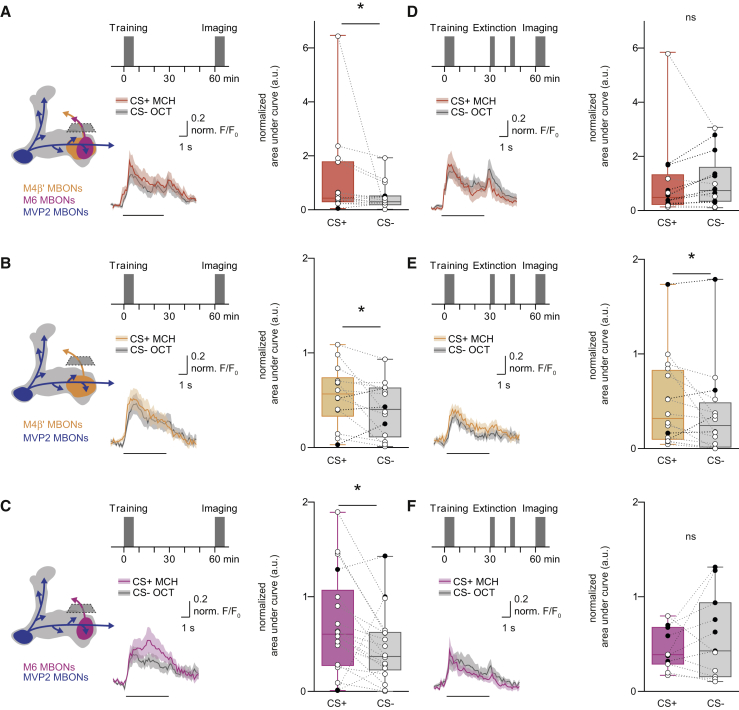
Aversive and Extinction Memories Are Integrated in M6 Neurons (A) Imaging plane and protocol. Aversive conditioning increases CS+ response in the axon of M4β′/M6 MBONs. (B and C) Potentiated response to CS+ evident in M4β′ (B) and M6 (C). (D) Extinction protocol. Extinction nullifies training-induced increase in CS+ response in M4β′/M6 axons. (E) Training-induced potentiation of CS+ response in M4β′ axon survives extinction. (F) Extinction nullifies training-induced increased CS+ response in M6 axon. Odor-evoked activity traces show mean (line) with SEM. (shadow). Black line, 5-s odor during imaging phase of the experiment. Paired measurements are the same as those used in Figure 3. Asterisks, significant difference between CS− and CS+ responses. See also Figures S5 and S7 and Table S1.

**Figure S5:**
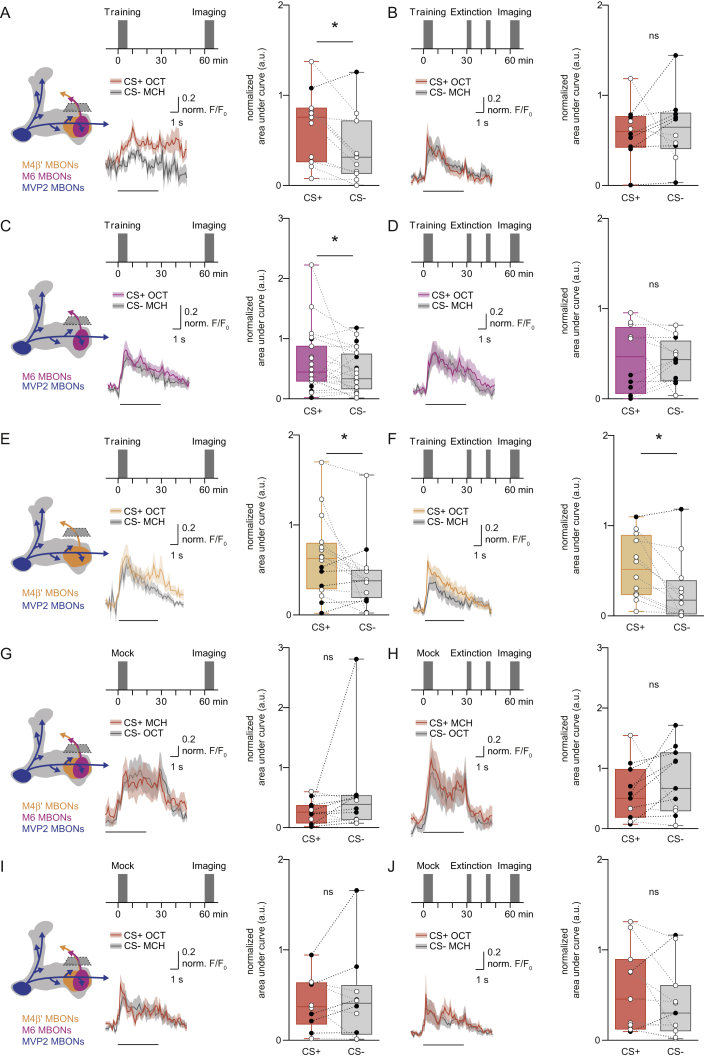
Aversive and Extinction Memories Are Integrated within M6 Neurons, Related to Figure 6 (A and B) Complementary experiment to Figures 6A and 6D. (A) Imaging plane and training protocol under the microscope. CS+ is OCT. Aversive conditioning increases CS+ odor response of axonal segment of M4β′ and M6 neurons. (B) Extinction protocol under the microscope. Aversive memory extinction nullifies the training-induced increase in CS+ odor response measured in axonal segment of M4β′/M6 neurons. (C and D) Complementary experiment to Figures 6C and 6F. (C) The potentiated response to the CS+ after aversive conditioning is evident in the axonal segment of M6 neuron. (D) Consistent with Figure 6F, extinction of aversive memory for OCT nullifies the training-induced increased CS+ odor response in the M6 axon. (E–H) Mock conditioning with OCT does not change odor responses measured in axonal segments of M4β′ and M6 neurons. Odor-evoked activity traces show mean (solid line) with SEM. (shadow). Black line, 5 s odor presentation. Paired measurements from individual flies displayed either as black (CS+ response < CS- response) or white (CS+ response > CS- response) dots. Asterisks, significant difference between averaged CS- and the CS+ response.

Flies trained and not subjected to the extinction protocol showed the previously reported (Owald et al., 2015) aversive memory trace of a relative potentiation of the CS+ odor response measured in the mixed neurites of the M4β′ and M6 neurons (Figure 6A, VT1211-GAL4). Measuring from M4β′ and M6 neurons individually showed that the potentiated CS+ response was present in both M4β′ and M6 neurons (Figures 6B and 6C; M4β′, R39A05-GAL4 and M6, R66C08-GAL4). However, following extinction, the increased CS+ response remained in the M4β′ neurite, but was no longer evident in the M6 neurite (Figures 6D–6F). These effects were also observed when the reciprocal odor was used as CS+, but not after mock training (Figures S5A–S5J). We therefore propose that the aversive and extinction memories are integrated within the M6 neurons, whose activity determines the robustness of the expression of conditioned avoidance behavior.

**Figure S6:**
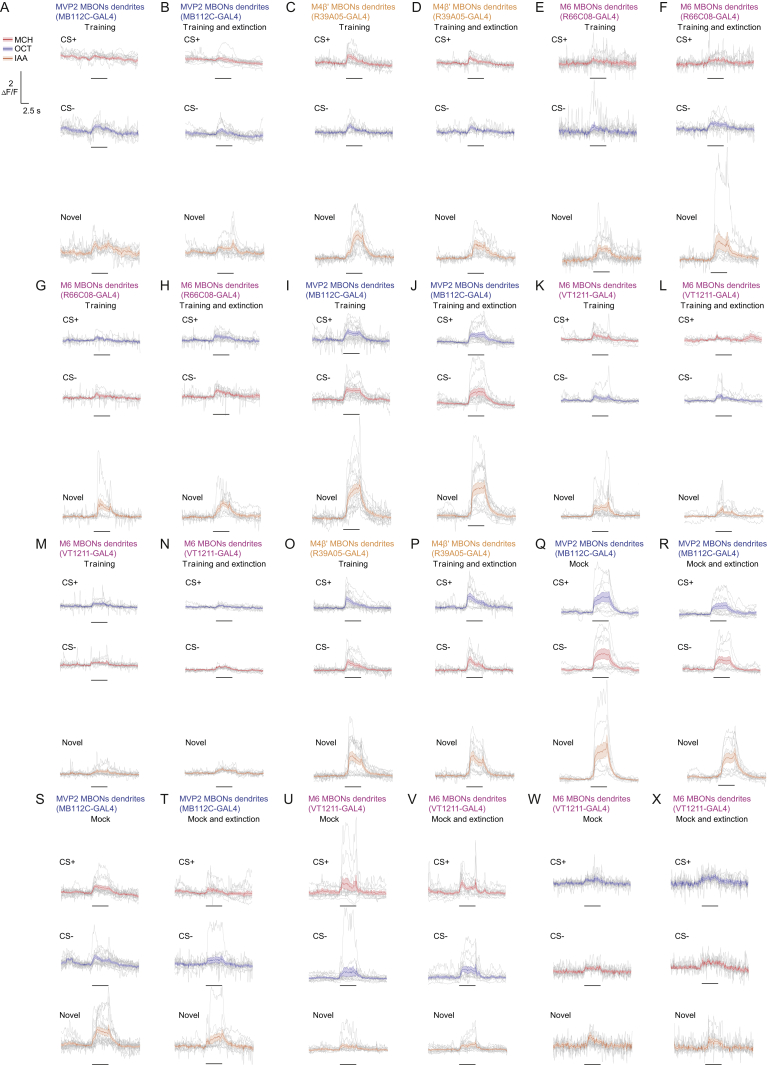
Parallel Memory Traces Form When Aversive Memory Is Extinguished, Related to Figures 3 and S3 (A–X) All imaging traces for odor responses to the CS+, the CS- (either OCT, blue, or MCH, red) or the novel odor (IAA, orange) for the experiments in the order as they are depicted in Figures 3 and S3. Individual traces (gray), the mean (colored solid line) and the SEM (shadow) are displayed. Black line represents 5 s odor presentation during the imaging phase of the experiment.

**Figure S7:**
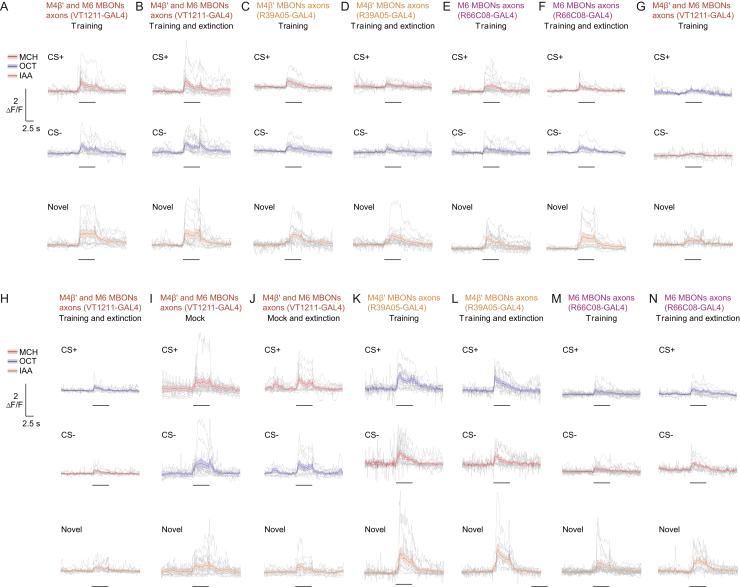
Aversive and Extinction Memories Are Integrated within the M6 Neurons, Related to Figures 6 and S6 (A–N) All imaging traces for odor responses measured in M6 axons to the CS+, the CS- (either OCT or MCH) or the novel odor (IAA) for the experiments in the order as they are depicted in Figures 6 and S6. Individual traces (gray), the mean (colored solid line) and the SEM (shadow) are displayed. Black line represents 5 s odor presentation during the imaging phase of the experiment.

## Discussion

Extinction was first described by Pavlov (1927) in his experiments with dogs. Although extinction is broadly believed to result from new inhibitory learning, rather than erasure of the original memory (Myers and Davis, 2007, Herry et al., 2010), the underlying neural mechanisms have remained elusive. In this study, we describe how competing memories arise and are integrated to extinguish aversive memory in *Drosophila*.

### How Does the Omission of Punishment Drive New Learning?

Extinction of aversive memory required PAM dopaminergic neurons during the period of odor re-exposure. Some of these DANs provide teaching signals when flies are trained with odor and sugar or water reward (Liu et al., 2012, Burke et al., 2012, Lin et al., 2014). Importantly, sugar reward learning mediated by these DANs induces relative depression of CS+ odor-evoked responses in M4β′/M6 MBONs (Owald et al., 2015), which we also observed following extinction of aversive memory. Since reduced odor-driven activity in M6 MBONs is enough to convert odor avoidance behavior into attraction (Owald et al., 2015, Barnstedt et al., 2016), plasticity of aversive memory extinction can be considered to be appetitive. These results together suggest that absence of predicted punishment is coded in the fly brain in a similar way to positive experience. But how can lack of punishment lead to a potential reward signal?

**Figure 7:**
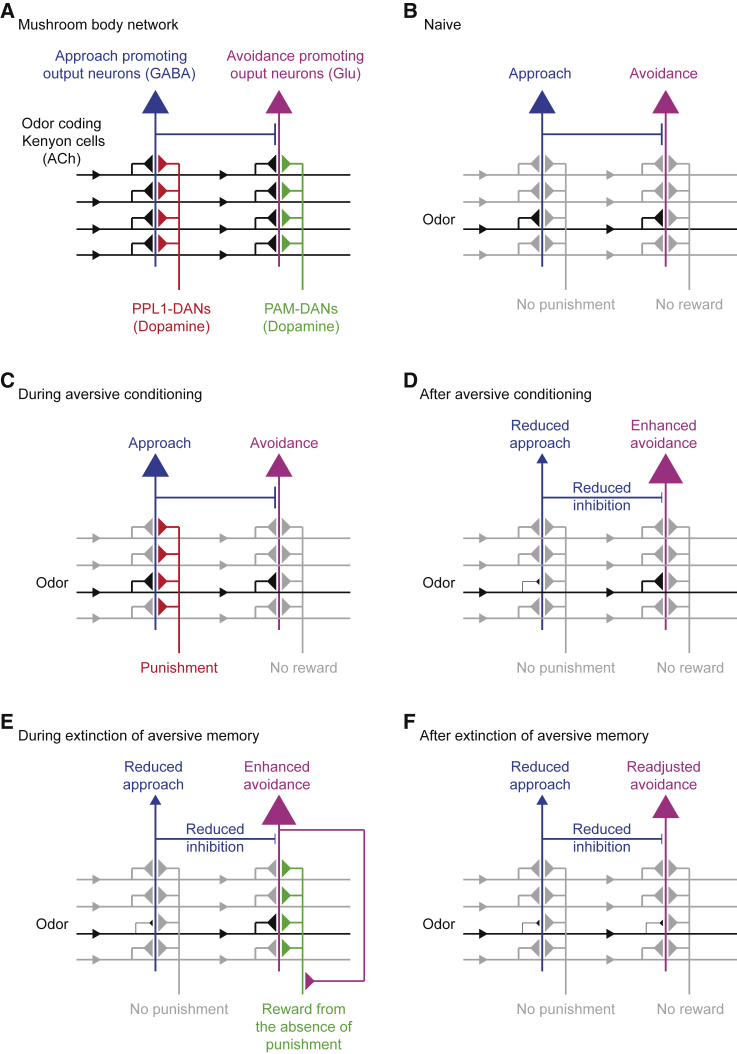
Model of Extinction: Aversive Memory Expression Is Limited by Competition with a Parallel Extinction Memory of Opposite Valence (A) Individual DANs from the PPL1 and PAM clusters innervate distinct mushroom body lobe compartments. PPL1-DANs (red) provide teaching signals during aversive conditioning and PAM-DANs (green) for appetitive conditioning. Each compartment, innervated by a particular DAN also houses dendrites of a corresponding MBON, which are GABAergic (blue), glutamatergic (magenta), or cholinergic (not shown). MBONs receive excitatory acetylcholine from odor coding KCs (black). Terminals of PPL1 DANs overlap dendrites of MBONs promoting approach behavior (blue), whereas PAM DANs overlap MBONs directing avoidance (magenta). MBONs drawn are valence-coding MBONs described to harbor traces of aversive or appetitive memory (Owald et al., 2015, Perisse et al., 2016). (B) When a naive fly detects neutral odor, odor-specific KCs (black) drive an equally weighted network of approach and avoidance promoting MBONs. This balanced network configuration does not promote directed behavior. (C) During aversive conditioning, CS+ induced activity in KCs and downstream MBONs coincides with activity of PPL1 DANs, leading to compartment restricted synaptic depression between odor-activated KCs and respective MBON. (D) Following aversive conditioning, CS+ drive to approach MBONs is reduced (smaller triangle) and as result of reduced odor-specific MVP2-mediated feedforward inhibition, CS+ drive to avoidance promoting M4β′/M6 is also potentiated. (E) During extinction, learned configuration of the MBON network favors CS+ activation of avoidance promoting MBONs, which, in turn, drives appetitively reinforcing γ5 PAM DANs. Coincidence of CS+ during extinction and γ5 DAN activity depresses odor-activated KC synapses onto M6 MBONs. (F) After extinction, reduced CS+ drive to avoidance coding M6 (smaller triangle) partially compensates for the network potentiation of M6 neuron response induced during initial aversive training.

Previous data and those presented here suggest that aversive learning reconfigures the MBON network into a state primed to preferentially drive a reward teaching signal, when the flies re-experience trained odor without punishment (Figure 7). Prior work, reproduced here, showed that aversive learning depresses conditioned odor drive to the KC-MVP2 MBON pathway, that favors approach behavior (Hige et al., 2015, Perisse et al., 2016). Furthermore, like the role for disinhibition in mice (Letzkus et al., 2015), aversive learning reduces MVP2-mediated feedforward inhibition in the network and thereby also indirectly potentiates M4β′/M6 MBON odor responses that drive avoidance behavior. Since some avoidance directing MBONs can provide recurrent input to PAM DANs (Owald et al., 2015, Aso et al., 2014b, Cohn et al., 2015), odor re-exposure after aversive learning should preferentially drive a positive teaching signal via these MBONs. When directly triggered, glutamatergic M4β′ and M6 neurons selectively activated DANs releasing dopamine in the γ5 compartment. Finding that extinction induced a corresponding depression of conditioned odor drive to M6 neurons is therefore also consistent with the previously trained odor activating γ5 DANs, to direct odor-specific plasticity at KC-M6 synapses.

We do not know whether extinction-relevant γ5 DANs are the same as those providing water or sugar reward-teaching signals. Despite our expectations, we were unable to observe increased odor-evoked activity in γ5 DANs after aversive learning, using GCaMP6m. R58E02-GAL4-labeled γ5 DANs exhibited robust oscillatory activity (data not shown), which impeded reliable recording of odor-evoked events. Some γ5 DANs may oscillate and others be cue evoked, but we currently lack the genetic tools to direct transgene expression to meaningful subsets. Nevertheless, there are between 8 and 21 γ5 DANs (Aso et al., 2014a) and γ5 presynaptic innervation within that MB compartment may be further segregated (Huetteroth et al., 2015). If individual γ5 DANs have input and output specificity, different KC-M6 synapses along the same odor-activated KC would be modified by sugar reward learning and aversive memory extinction, thereby expanding the coding range within the KC-MBON network. Nevertheless, this level of potential synaptic specificity of reward learning and extinction would still generate a similar odor-specific depression when recording broad odor-evoked signals from M6 dendrites. Although anatomical specificity is appealing and not at odds with our current and prior data (Owald et al., 2015), it will be essential to determine how individual γ5 DANs operate and analyze KC-M6 dendritic plasticity at higher resolution.

Without knowing the specific location of extinction-driven synaptic plasticity, our model predicts that if punishment does not follow conditioned odor presentation, extinction plasticity triggered at the KC-M6 MBON junction readjusts the balance in the MBON network. Whereas if shock were to follow, extinction plasticity would be offset by additional modification made to the site of the original aversive memory. We assume an opposite scenario underlies the extinction of appetitive memory, which is initially coded as depression of conditioned odor drive to avoidance directing MBONs (Owald et al., 2015, Felsenberg et al., 2017). Re-exposing flies to the conditioned odor, without sugar, neutralizes odor driven approach (Tempel et al., 1983). However, this process instead required aversively reinforcing DANs, some of which are functionally connected to approach directing MBONs. Omission of predicted reward therefore appears to be coded as aversive experience (Felsenberg et al., 2017). Taken with data here, we propose that DAN-driven formation of a competing memory of opposite valence is a general, and likely conserved (Pan et al., 2013), feature of memory extinction.

Prediction error, an unexpected change in reward or punishment contingency, has a strong theoretical and experimental foundation in mammalian dopaminergic neurons (Rescorla and Wagner, 1972, Schultz et al., 1997, Steinberg et al., 2013, Pan et al., 2013, Matsumoto and Hikosaka, 2009, Bromberg-Martin et al., 2010). However, it is not clear how errors are registered and how dopaminergic activity alters the underlying network. By coding valence of learning as a particular skew in the MBON network, the fly can use opposing arms of the DAN system to keep track of when expected contingencies between odors and positive or negative events are not met. Such a model predicts that odors that are learned to be avoided will preferentially trigger appetitively reinforcing DANs if punishment does not follow, whereas odors learned to be approached will more strongly activate aversive DANs and be registered as bad, if the expected reward is omitted.

### Parallel Memories Co-exist and Compete to Direct Odor-Driven Behavior

We observed physiological traces of the original aversive memory, and new extinction memory in different nodes of the MBON network at the same time after training. An aversive memory trace measurable in the dendrites of MVP2 neurons survived extinction, while a new extinction trace arose in the odor responsiveness of M6 neurons. Although functional imaging suggests that the change in relative odor drive from KCs to MVP2 MBONs that accompanies aversive learning remains after extinction, we cannot be sure that it results from the same unaltered synaptic or neural mechanism.

Flies simultaneously form parallel memories of opposite valence, if trained with odor and sugar laced with bitter taste. These separate aversive and appetitive memories compete to guide either learned odor avoidance or approach behavior (Das et al., 2014). Since aversive memory followed by extinction is equivalent to sequential formation of parallel memories, it follows that a new extinction memory written in the KC-M6 MBON connection by γ5 DANs, can partially neutralize behavioral expression of the original aversive memory, formed at the KC-MVP2 junction. Since multiple MBON pathways (e.g., MVP2 and V2α) are modified by aversive learning (Séjourné et al., 2011, Owald et al., 2015, Hige et al., 2015, Perisse et al., 2016), but only the KC-M6 junction is modified by extinction (not KC-M4β′), an imbalanced number of plastic connections might account for the partial nature of aversive memory extinction.

The apparent stability of learning induced changes in odor-evoked activity in MVP2 neurons after extinction, taken with our retraining experiments indicate that flies can accumulate information across training, extinction, and retraining trials. We propose that retention of learned information following extinction is a fundamental feature of a memory network. Combining supporting and conflicting information from consecutive experience is certainly a prerequisite for more complex probabilistic learning.

### MVP2 Neurons Make Different Types of Feedforward Inhibitory Synapses

MVP2 neurons innervate multiple compartments of the MB and appear to make different connections with vertical and horizontal lobe MBONs (Perisse et al., 2016). Ultrastructure shows that an MVP2 neuron forms distinct synaptic connections with M4β′ and M6 MBONs. Whereas MVP2 makes large bouton-type synapses onto M4β′ distal dendrites, MVP2 forms *en passant* synapses along M6 primary neurites. These connections are reminiscent of those made by unique types of mouse GABA-ergic neurons (Krabbe et al., 2018).

### Aversive and Extinction Memories Are Integrated in the M6 Neurons

Recent EM reconstruction of the larval MB wiring diagram described connections between MBONs, and convergence neurons pooling collections of MBON inputs (Eichler et al., 2017). We found that aversive and extinction memories are already integrated within the MBON network and specifically in M6 neurons, that promote avoidance. The learning induced potentiated odor-response in M6, resulting from reduced MVP2 mediated inhibition, appeared nullified by addition of odor-specific depression of the KC-M6 connection. This suggests that extinction memory can suppress expression of the original aversive memory and consequently learned odor avoidance behavior.

It is not known how *Drosophila* appetitive memories are countered by their corresponding extinction memory to suppress conditioned approach. At present the MBON network architecture looks more complex than a straightforward “winner-takes-all” scenario involving direct reciprocal inhibitory connections between approach and avoidance directing pathways.

### Depending on Time, Extinguished Memory May Spontaneously Recover

We exclusively studied extinction soon after training. Prior studies in flies and other animals suggest processes might differ at later times (Hirano et al., 2016, Myers et al., 2006, Eisenberg and Dudai, 2004). Given expression of longer-term memories is apparently more reliant on αβ than γ KCs (Yu et al., 2006, Krashes and Waddell, 2008, Bouzaiane et al., 2015), it is possible odor re-exposure at later times will drive a different imbalanced MBON network configuration than that earlier on. In this case, other appetitively reinforcing DANs, and plasticity at different KC-MBON junctions, might be required to acquire a competing extinction memory at that time.

Sometimes extinguished memories spontaneously recover with time, consistent with a new memory temporarily suppressing previous learned behavior (Rescorla, 2004, Bouton et al., 2006). In *Drosophila*, spontaneous recovery of extinguished aversive memory is time dependent. Memories extinguished 2 days after training remain low for 4 days, whereas those extinguished at 5 days recover 4 days later (Hirano et al., 2016). Recovery of extinguished memories could be accompanied by loss of odor-specific plasticity in KC-M6 dendrites. Furthermore, the ability of extinguished memories to recover might result from the relative strength of KC-MBON connections in which the original aversive memory resides, and the extinction memory is formed, at the time the fly re-encounters the CS+ without punishment.

### Could Extinction Function Similarly in Mammals?

Some reward-activated mammalian DANs (Schultz et al., 1997) also respond to absence of an expected aversive stimulus (Matsumoto and Hikosaka, 2009, Bromberg-Martin et al., 2010). Therefore, fear extinction also could be triggered by appetitively reinforcing DANs (Luo et al., 2018). Acquisition and extinction of fear memory involves plasticity in basolateral amygdala (BLA) (Bocchio et al., 2017), which contains distinct neural paths for fear and reward memories (Shabel and Janak, 2009, Belova et al., 2007, Redondo et al., 2014, Gore et al., 2015, Namburi et al., 2015, Beyeler et al., 2016, Kim et al., 2016). Perhaps an analogous arrangement of parallel competing memories (Grewe et al., 2017), driven by teaching signals (Saunders et al., 2018) from BLA-projecting DANs (Lammel et al., 2014), extinguishes mammalian fear.

### A Numerically Simple, yet Functionally Efficient, Neural Network?

An early mechanistic study of *Drosophila* extinction concluded that aversive learning and its extinction both occur within the same subset of KCs (Schwaerzel et al., 2002). In addition, the authors proposed extinction involved intracellular antagonism with cAMP signaling that is required for memory formation. Our data suggest initial aversive learning and subsequent extinction are coded as consecutive learning events within the same odor-activated KCs. However, two parallel memories are formed within anatomically separate output compartments of the same KCs where they synapse onto different MBONs. Learned behavior is therefore extinguished as a result of intercellular antagonism within the output layer of the MB network. This process is likely reliant on the extended architecture of KCs that separates KCs’ primary sensory input layer in the MB calyx from a compartmentalized error adjustment layer in the lobes. Activity in populations of KCs therefore represents specific odors, whereas associated values, such as unexpected shock and absence of predicted shock, can be independently and locally assigned to odors by altering the weights of synapses in different output compartments from the same KCs.

## STAR★Methods

### Key Resources Table

**Table:** 

REAGENT or RESOURCE	SOURCE	IDENTIFIER
**Antibodies**		

DsRed Polyclonal	Clonetech Laboratories	RRID: AB_10013483
Anti-GFP	Abcam	RRID: AB_300798

**Chemicals, Peptides, and Recombinant Proteins**		

N-Tris	Sigma-Aldrich	Cat#T5691
NaCl	Sigma-Aldrich	Cat#S7653
KCl	Sigma-Aldrich	Cat#P9333
NaHCO_3_	Sigma-Aldrich	Cat#S6297
NaH_2_PO_4_	Sigma-Aldrich	Cat#S8282
CaCl_2_	Sigma-Aldrich	Cat#21115
MgCl2	Sigma-Aldrich	Cat#M1028
Trehalose	Sigma-Aldrich	Cat#T9531
Glucose	Sigma-Aldrich	Cat#G7528
Sucrose	Sigma-Aldrich	Cat# S0389
Mineral Oil	Sigma-Aldrich	Cat#M5904
4-methylcyclohexanol (98%)	Sigma-Aldrich	Cat#218405
3-octanol (99%)	Sigma-Aldrich	Cat#153095
Isopentyl acetate (99%)	Sigma-Aldrich	Cat#306967

**Experimental Models: Organisms/Strains**		

*Drosophila*: VT1211-GAL4	*Vienna Drosophila* RNAi Center; Owald et al., 2015	RRID:VDRC: 202324
*Drosophila*: MB112C-GAL4	Bloomington *Drosophila* Stock Center; Aso et al., 2014a, Aso et al., 2014b	RRID:BDSC_68263
*Drosophila*: R66C08-GAL4	Bloomington *Drosophila* Stock Center; Owald et al., 2015	RRID:BDSC_49412
*Drosophila*: R39A05-GAL4	Bloomington *Drosophila* Stock Center; Jenett et al., 2012	RRID:BDSC_50033
*Drosophila*: R48B04-GAL4	Bloomington *Drosophila* Stock Center; Jenett et al., 2012	RRID:BDSC_50347
*Drosophila*: R58E02-GAL4	Bloomington *Drosophila* Stock Center; Jenett et al., 2012	RRID:BDSC_41347
*Drosophila*: VT1211-LexA	this paper	N/A
*Drosophila*: MB504B-GAL4	Bloomington *Drosophila* Stock Center; Aso et al., 2014a, Aso et al., 2014b	RRID:BDSC_68329
*Drosophila*: UAS-GCaMP6m	Bloomington *Drosophila* Stock Center; Chen et. al., 2013	RRID:BDSC_42748
*Drosophila*: UAS-GCaMP6f	Bloomington *Drosophila* Stock Center; Chen et. al., 2013	RRID:BDSC_42747
*Drosophila*: UAS-*Shi*^ts1^	Kitamoto 2001	N/A
*Drosophila:* lexAop-CsChrimson-tdTomato, UAS-GCaMP6f	Hoopfer et al., 2015	N/A

**Software and Algorithms**		

Fiji	NIH; Schindelin et al., 2012	http://fiji.sc/
MATLAB R2017b	The Mathworks, Natick, MA	https://www.mathworks.com/products/matlab.html
GraphPad Prism 6	GraphPad Software, La Jolla, CA	https://www.graphpad.com/scientific-software/prism/
Adobe Illustrator CC	Adobe Systems, San Jose, CA	https://www.adobe.com/uk/products/illustrator.html
TrakEM2	Cardona et al., 2012	https://imagej.net/TrakEM2
Blender	Blender Online Community	https://www.blender.org/
R	R Development Core Team, 2008	http://www.R-project.org/
Python	Python Software Foundation	https://www.python.org/
PyMaid	Philipp Schlegel	https://github.com/schlegelp/PyMaid
Rcatmaid	Jefferis lab, Albert Cardona, Philipp Schlegel	http://jefferis.github.io/rcatmaid/
CATMAID	Saalfeld et al., 2009, Schneider-Mizell et al., 2016	https://catmaid.readthedocs.io/en/stable/index.html#
Dendrogram code	Markus Pleijzier	https://github.com/markuspleijzier/AdultEM/tree/master/Dendrogram_code
Graphviz	Gansner and North, 2000	https://www.graphviz.org
NetworkX	Hagberg et al., 2008	https://networkx.github.io/
ScanImage 3.8 software	Pologruto et al., 2003	https://vidriotechnologies.com/

### Contact for Reagent and Resource Sharing

Further information and requests for resources and reagents should be directed to and will be fulfilled by the Lead Contact, Scott Waddell (scott.waddell@cncb.ox.ac.uk)

### Experimental Model and Subject Details

#### Fly strains

All *Drosophila melanogaster* strains were reared at 25°C and 40%–50% humidity on standard cornmeal-agar food in 12:12 h light:dark cycle. 2-9 day old adult flies were used. Canton-S flies were used as wild-type. Transgenes were expressed with GAL4 lines produced by the Janelia FlyLight (Jenett et al., 2012) and Vienna Tiles Projects (Tirian and Dickson, 2017) and are described; R58E02-GAL4 (Liu et al., 2012), MB504B-GAL4 (Aso et al., 2014a), MB112C-GAL4 (Aso et al., 2014b), R66C08-GAL4 and VT1211-GAL4 (Owald et al., 2015), R48B04-GAL4 (Huetteroth et al., 2015), R39A05-GAL4 (Jenett et al., 2012). For behavioral experiments UAS-*Shi*^ts1^ (Kitamoto, 2001) was expressed under the control of the respective GAL4 driver. For the imaging experiments UAS-GCaMP6m and UAS-GCaMP6f (Chen et al., 2013), and the artificial activation experiments lexAop-CsChrimson- tdTomato,UAS-GCaMP6f (Hoopfer et al., 2015), were expressed with the respective GAL4 and LexA drivers.

### Method Details

#### Behavioral experiments

Male flies from the GAL4 lines were crossed to UAS-*Shi*^ts^ females and 4 to 9-day-old mixed-sex progeny were tested together in all experiments. Approximately 80 - 100 flies were placed in a 25 mL vial containing standard food and a 20 × 60 mm piece of filter paper for 14–22 hours before behavioral experiments. Odors used in all experiments were 4-methylcyclohexanol (MCH) and 3-octanol (OCT) diluted in mineral oil. An odor dilution of ∼1:10^4^ or 1:10^6^ was used for all experiments except in the pre-exposure experiments where odor dilutions were ∼1:10^3^ (specifically, 7 μL OCT, or 12 μL MCH in 8 mL mineral oil), ∼1:10^4^ (specifically, 300 nL OCT, or 800nl - 1.6 μL MCH in 8 mL mineral oil) and ∼1:10^6^ (specifically, 7 nL OCT, or 12 nL MCH in 8 mL mineral oil). All experiments were performed at 23°C and 55%–65% relative humidity. Temperature was only raised to the restrictive 30-33°C, during the odor reactivation or test phase of the *Shi*^*ts1*^ experiments.

Aversive olfactory conditioning in the T-maze was conducted as previously described (Tully and Quinn, 1985, Perisse et al., 2016). Groups of flies were exposed to a first odor for 1 min (the conditioned stimulus+, CS+) paired with twelve 90 V electric shocks at 5 s intervals. Following 45 s of clean air, a second odor (the conditioned stimulus-, CS−) was presented for 1 min without shock. Memory was subsequently assessed by testing flies for their odor-preference between the CS- and the CS+ in a T-maze (2 min). Performance Index was calculated as the number of flies in the CS+ arm minus the number in the CS- arm, divided by the total number of flies (Tully and Quinn, 1985). MCH and OCT, were alternately used as CS+ or CS- and a single sample, or n, represents the average performance score from two reciprocally trained groups.

We optimized a published extinction protocol (Schwaerzel et al., 2002) so that the concentration of odor used in the re-exposure experiments did not alter odor-driven behavior when naive flies received successive presentations (Figure S1A). During the odor re-exposure phase either the CS+ or CS- odor was presented for 1min in the training tube. The inter-trial interval (ITI) refers to the time between the end of the preceding trial to the start of the next trial. The retraining trial (Figure S1C) consisted of a single 1 min CS+ pairing with twelve 90 V electric shocks at 5 s interval.

#### Functional connectivity experiments

These experiments were conducted under a two-photon microscope (Scientifica), essentially followed our previously published protocol (Barnstedt et al., 2016). 6-7 day old female flies were housed on standard food supplemented with 1mM retinal for 1-2 days. Fly brains were removed from the head capsule and adhered to a polylysine coated coverslip bathed in carbogenated (95% O_2_, 5% CO_2_) buffer solution (103 mM NaCl, 3 mM KCl, 5mM N-Tris, 10 mM trehalose, 10 mM glucose, 7mM sucrose, 26 mM NaHCO_3_, 1mM NaH_2_PO_4_, 1.5 mM CaCl_2_, 4mM MgCl_2_, osmolarity 275 mOsm, pH 7.3) after dissection in cold calcium-free buffer. For optogenetic light activation a high-power LED (Mulitcomp OSW-6338, 630nm) was relayed onto the specimen via a 50mm diameter lens with focal length of 60mm filtered through a 632/10 bandpass filter (Edmund Optics). The power at the specimen was measured to be 0.85 mW mm^2^ and the LED was triggered by a microcontroller (Arduino MEGA). After identification and focusing on the targeted field of view, brains were let rest for 5 min. 10 s after recording the baseline fluorescence (F), 10 ms light pulses were delivered at 40 Hz for a total of 500 ms. Fluorescence was excited using 140-fs pulses, 80MHz repetition rate, centered on 910 nm generated by a Ti-Sapphire laser (Chameleon Ultra II, Coherent) and images of 256 X 256 pixels were acquired at 5.92Hz, controlled by ScanImage 3.8 software (Pologruto et al., 2003). Processes of dopaminergic neurons were imaged at the level of the tip of the horizontal mushroom body lobe and signals were assigned to discrete DANs that innervate the β′2, γ4 and γ5 compartments by manually drawing a region of interest (ROI) in the relevant areas. Images were manually segmented and further analyzed using customized MATLAB scripts. F_0_ was defined as the mean F from the first 9 s of baseline recording. ΔF/F_0_ was compared between 1 s before the stimulation with 1 s after stimulation onset, using a paired t test.

#### Aversive conditioning under the microscope

3-8 day old adult female and male flies were immobilized on ice and mounted in a custom made chamber, allowing free movement of the antennae and legs. The head capsule was opened under room temperature carbogenated buffer (see section above) and the fly, in the recording chamber, was placed under the Two-Photon microscope. A constant air stream, carrying vapor from mineral oil solvent (air), was applied and an electrifiable grid was raised from below until the fly’s legs made contact. Flies were trained and re-exposed to odors under the microscope using essentially the same regimens and odor concentrations as those in the behavioral experiments. An odor stream was added to the air for 1 min (CS+) while twelve 90 V electric shocks were delivered to the fly’s legs. The first electric shock arrives 1.2 s after the onset of the CS+ odor. Following 45 s of air, a CS- odor was added for 1 min to the clean air stream and presented to the fly without electric shock. Trained flies were either re-exposed to two CS+ trials (15 min ITI) without the electric shock, or were left untreated. After training, flies in the custom chamber were removed from the microscope and rested until the odor re-exposure, or test phase of the experiment. For odor re-exposure flies were placed into the airstream for 30 s followed by two 1 min exposures to the CS+ (with a 15 min ITI). The carbogenated buffer was changed before each re-exposure phase, or every 30 min for the flies not re-exposed to odor. To control for odor exposure effects an independent set of flies was subjected to mock training: the same odor regimen as in training but no electric shock was applied. One hour after training, or mock training, GCaMP responses to the CS+, the CS- and a novel odor were measured in the relevant MBONs. The flies were sequentially exposed to the CS+, CS- and a novel odor, isoamyl acetate (IAA; 1:10^6^ odor concentration) interspersed by 30 s of air (Owald et al., 2015, Perisse et al., 2016). To image the dendritic field of MVP2 or the axonal segments of the M4β′ and M6 (and M4β′/M6 together) neurons, processes in one hemisphere of the brain were selected. To measure responses in the M4β′ and M6 (and M4β′/M6) dendrites, signals were simultaneously acquired from both hemispheres and averaged responses were analyzed.

Fluorescence was excited using ∼140 fs pulses, 80 MHz repetition rate, centered on 910 nm generated by a Ti-Sapphire laser (Chameleon Ultra II, Coherent). Images were acquired with a Two-Photon microscope (Scientifica) with a 403, 0.8 NA water-immersion 40X objective, controlled by ScanImage 3.8 software (Pologruto et al., 2003). Odors were delivered using a custom-designed system (Shang et al., 2007). Shock voltage and delay was controlled by a DS2A Isolated Constant Voltage Stimulator (Digitimer, Hertfordshire, UK) and a DG2A Train/Delay Generator (Digitimer), respectively.

For analysis, two-photon fluorescence images were manually segmented using Fiji (Schindelin et al., 2012). Movement of the animals was small enough such that images did not require registration. For subsequent quantitative analyses, custom Fiji and MATLAB scripts were used. The baseline fluorescence, F_0_, was defined for each stimulus response as the mean fluorescence F from 2 s before and up to the point of odor presentation. F/F_0_ accordingly describes the fluorescence relative to this baseline. The area under the curve (AUC) was measured as the integral of F/F_0_ during the 5 s odor stimulation. To account for variance between individual flies, the responses of the CS+ and CS− were normalized to the response to IAA. Each AUC was divided by the IAA AUC from the respective trial and individual fly. Boxplots show the 25^th^ - 75^th^ percentiles (box), the median (line) and the minimum and maximum (whiskers) values for the normalized area under the curve of the responses during the odor presentation.

#### Light microscopy

The signal of the GFP and RFP proteins was boosted using anti-GFP and anti-DsRed antibodies as described (Perisse et al., 2016). Imaging was performed using a ZEISS Laser Scanning Microscope (LSM) 880 equipped with a fast Airyscan detection unit (Carl Zeiss AG, Oberkochen, Germany). We used a high NA oil immersion alpha Plan-Apochromat 63X/1.46 Oil Corr M27 objective with Immersol 518F immersion media (n_e_ = 1.518 at 23°C). In line scanning confocal mode we adjusted laser power, pixel offset and gain to avoid clipping at zero signal and saturation. This optimization was performed in the top z-section of the acquisition, which is usually the brightest of the stack. In Airyscan mode the beam was aligned on a cropped high intensity ROI and remained fixed over the entire acquisition. Airyscan reconstructions were made in Zeiss ZEN 2.3 (blue edition) using automated regularisation selection.

#### Neuron reconstruction - ‘tracing’

Neurons were traced in a serial section transmission electron microscopy (ssTEM) volume of a full adult female *D. melanogaster* brain (FAFB) (Zheng et al., 2018) using CATMAID, a web-based software for collaborative neural circuit reconstruction from large image datasets (https://catmaid.readthedocs.io/en/stable/) (Saalfeld et al., 2009, Schneider-Mizell et al., 2016). Consistent with previous studies (Eichler et al., 2017, Schlegel et al., 2016, Schneider-Mizell et al., 2016), tracing followed the centerline of a neuron’s profiles through the dataset to reconstruct neurite morphology and annotate synaptic sites. We used an iterative approach established and tested by Schneider-Mizell et al. (2016), where initial reconstruction is followed by a systematic proofreading by at least two experienced reviewers (> 500h of tracing experience).

MBON identification: MBONs were located by sampling downstream of previously identified KC synapses in the respective mushroom body lobe compartments. Their identity was confirmed by comparison with light level data (Aso et al., 2014b).

Synapse annotation: Synaptic sites were identified based on three, previously described criteria (Prokop and Meinertzhagen, 2006) and reviewed as above: an active zone with (1) T-bar(s) and (2) surrounding vesicle cloud, and (3) a synaptic cleft to which all postsynaptic neurons must have access.

In *Drosophila*, presynapses have been found on fine axonal processes (Schneider-Mizell et al., 2016), boutons (Butcher et al., 2012), and other neurites that are neither in the dendritic nor the axonal field. Post-synapses have been found on large or fine dendritic processes and fine spine like twigs that are shorter than 3μm (Schneider-Mizell et al., 2016). M4β′ M6R and M6L were reconstructed the same way to maintain consistency in the placement of synapses. Schneider-Mizell et al. (2016) estimated that the tracing approach employed typically finds 99.8% of all pre- and 91.7% of all post-synapses. The probability of identifying false-positive post-synapses is 2.2% and negligible for presynapses. Since all synaptic sites on the MVP2 axon and M4β′ and M6 dendrites were annotated, we can estimate the upper and lower bounds of the number of synapses between MVP2 and M4β′ or M6 neurons (Note only integer numbers of synapses are expected):

**Table:** 

	Found	Lower Bound	Upper Bound
MVP2- > M6R	17	16.6	18.4
MVP2- > M6L	16	15.6	17.3
MVP2- > M4β'	47	46	50.8

Our error margins are likely smaller than those listed above, because the respective neurites of all neurons were more extensively reviewed than the agreed standard.

Reconstructed neurons were visualized using Blender 3D, an open-source 3D software (https://www.blender.org/). Neuron data from CATMAID were imported and shaded by Strahler order using an existing CATMAID plugin for Blender (https://github.com/schlegelp/CATMAID-to-Blender; Schlegel et al., 2016).

Volumetric reconstruction of synapse architecture was achieved by importing and annotating FAFB image data into ImageJ using the TrakEM2 plugin (Cardona et al., 2012). Reconstructions were exported for rendering to Blender 3D.

Analysis: All analyses were performed in R and Python using open-source software. PyMaid (https://github.com/schlegelp/PyMaid) and RCatmaid (http://jefferis.github.io/rcatmaid/; http://jefferis.github.io/elmr/) were used to interface with CATMAID servers and perform morphological analyses. Dendrogram representations of neural arbors were generated using new code (https://github.com/markuspleijzier/AdultEM/tree/master/Dendrogram_code) the graphviz library (https://graphviz.gitlab.io/; Gansner and North, 2000 via Python bindings provided by NetworkX, https://networkx.github.io/; Hagberg et al., 2008). M4β′ axonlets were defined as distal parts of neurites originating from the dendritic field, which made exclusively presynaptic connections. Axonlets were isolated and imported into Blender 3D using PyMaid. The root of the dendritic field was defined as the point at which the neuron’s main neurite branched into proximal dendrites and distal axon. Geodesic (along the arbor) distances between synapses and dendritic root were calculated using RCatmaid.

### Quantification and Statistical Analysis

Statistical analyses were performed in GraphPad Prism. All behavioral data were analyzed with an unpaired t test or a one-way ANOVA followed by a posthoc Tukey’s multiple comparisons test. No statistical methods were used to predetermine sample size. For the imaging experiments normalized responses were compared by a paired t test for normally distributed data, otherwise a Wilcoxon matched-pairs signed rank test was used for non-Gaussian distributed data. The respective statistical tests used, the n numbers and the p values can be found in the Table S1.

### Data and Software Availability

Customized MATLAB and Python scripts used in this paper are available upon request.

## Supplemental Information

Table S1. Experimental and Statistical Details, Related to Figures 1, 2, 3, 6, S1, S2, S3, S4, S5, S6, and S7 na, not applicable, n too low for normality test.
