# Supplementary material for: Integration of Parallel Opposing Memories Underlies Memory Extinction
Source: Cell. 2018 Oct 18;175(3):709–722.e15. doi: 10.1016/j.cell.2018.08.021 (PMC6198041; doi:10.1016/j.cell.2018.08.021)
Supplement: Table S1. Experimental and Statistical Details, Related to Figures 1, 2, 3, 6, S1, S2, S3, S4, S5, S6, and S7 — na, not applicable, n too low for normality test. [file mmc1.pdf]

Cell, Volume *175*

Supplemental Information

Integration of Parallel Opposing

Memories Underlies Memory Extinction

Johannes Felsenberg, Pedro F. Jacob, Thomas Walker, Oliver Barnstedt, Amelia J. Edmondson-Stait, Markus W. Pleijzier, Nils Otto, Philipp Schlegel, Nadiya Sharifi, Emmanuel Perisse, Carlas S. Smith, J. Scott Lauritzen, Marta Costa, Gregory S.X.E. Jefferis, Davi D. Bock, and Scott Waddell

Table 1. Statistical details related to Figure 1-6 and S1-S7

Experiment / Figure	group/treatment	n	normally distributed	statistical test	p value
Figure 1A	a) No extinction	6	na	ANOVA with Tukey's multiple comparisons test	ANOVA = 0.0025, Turkey's: a vs b = 0.99, a vs c = 0.02, a vs d = 0.04, b vs c = 0.01, b vs d = 0.03, c vs d = 0.98
	b) 2 CS+, 1 min ITI	10	yes		
	c) 2 CS+, 15 min ITI	10	yes		
	d) 5 CS+, 1 min ITI	10	yes		
Figure 1B	a) No extinction	7	na	ANOVA with Tukey's multiple comparisons test	ANOVA = 0.0025, Turkey's: a vs b = 0.97, a vs c = 0.003, b vs c = 0.004
	b) 2 CS-, 15 min ITI	7	na		
	c) 2 CS+, 15 min ITI	7	na		
Figure 1C	a) R58E02-GAL4 control CS- exposed	8	yes	unpaired t-test for comparision between same genotype but different treatment, ANOVA with Tukey's multiple comparisons test for comparision between different genotype, same treatment	ANOVA (CS-) = 0.0081, Turkey's: a vs c = 0.0292, a vs e = 0.0104, c vs e = 0.8768, ANOVA (CS+) = 0.0046, Turkey's: b vs d = 0.88, b vs f = 0.02, d vs f = 0.006, unpaired t-test: a vs b < 0.0001, c vs d = 0.02, e vs f = 0.4298
	b) R58E02-GAL4 control CS+ exposed	7	na		
	c) UAS-shits control CS- exposed	8	yes		
	d) UAS-shits control CS+ exposed	7	na		
	e) R58E02-GAL4; UAS-Shits1 CS- exposed	8	yes		
	f) R58E02-GAL4; UAS-Shits1 CS+ exposed	7	na		
Figure 1 D	a) MB504B-GAL4 control CS- exposed	6	na	unpaired t-test for comparision between same genotype but different treatment, ANOVA with Tukey's multiple comparisons test for comparision between different genotype, same treatment	ANOVA (CS-) = 0.8581, ANOVA (CS+) = 0.9763, unpaired t-test: a vs b = 0.001, c vs d = 0.01, e vs f = 0.03
	b) MB504B-GAL4 control CS+ exposed	6	na		
	c) UAS-Shits control CS- exposed	6	na		
	d) UAS-Shits control CS+ exposed	6	na		
	e) MB504B-GAL4; UAS-Shits1 CS- exposed	6	na		
	f) MB504B-GAL4; UAS-Shits1 CS+ exposed	6	na		
Figure 2 A and B	g 5	5	na	paired t-test	p = 0.0086
	g 5 control	4	na	paired t-test	p = 0.2664
Figure 2 C	g4	5	na	paired t-test	p = 0.2059
Figure 2 D	b'2p	4	na	paired t-test	p = 0.061
Figure 2 E	b'2m	4	na	paired t-test	p = 0.1805
Figure 2 F	a) VT1211-GAL4 control CS+ exposed	16	yes	ANOVA with Tukey's multiple comparisons test	ANOVA = 0.0108, Turkey's: a vs b = 0.99, a vs e = 0.02, b vs c = 0.02
	b) UAS-Shits1 control CS+ exposed	16	no		
	c) VT1211-GAL4; UAS-Shits1 CS+ exposed	16	yes		
	a) VT1211-GAL4 control CS- exposed	9	yes	ANOVA with Tukey's multiple comparisons test	ANOVA < 0.0001, Turkey's: a vs b = 0.88, a vs e < 0.0001, b vs c = 0.0003
	c) UAS-Shits1 control CS- exposed	9	yes		
	e) VT1211-GAL4; UAS-Shits1 CS- exposed	9	yes		
Figure 3A	MB112C-GAL4 dendrites training no extinction MCH as CS+	11	no	Wilcoxon matched-pairs signed rank test	p=0.0186
Figure 3B	R39A05-GAL4 dendrites training no extinction MCH as CS+	12	no	Wilcoxon matched-pairs signed rank test	p=0.8501
Figure 3C	R66C08-GAL4 dendrites training no extinction MCH as CS+	14	no	Wilcoxon matched-pairs signed rank test	p=0.6698
Figure 3D	MB112C-GAL4 dendrites training extinction MCH as CS+	15	no	Wilcoxon matched-pairs signed rank test	p=0.0125
Figure 3E	R39A05-GAL4 dendrites training extinction MCH as CS+	10	yes	paired t-test	p=0.3506
Figure 3F	R66C08-GAL4 dendrites training extinction MCH as CS+	12	yes	paired t-test	p=0.0019
Figure 6A	VT1211-GAL4 axons no extinction MCH as CS+	15	no	Wilcoxon matched-pairs signed rank test	p=0.0151
Figure 6B	R39A05-GAL4 axons training no extinction MCH as CS+	14	yes	paired t-test	p=0.0378
Figure 6C	R66C08-GAL4 axons training no extinction MCH as CS+	18	yes	paired t-test	p=0.0299
Figure 6D	VT1211-GAL4 axons training extinction MCH as CS+	14	no	Wilcoxon matched-pairs signed rank test	p=0.2166
Figure 6E	R39A05-GAL4 axons training extinction MCH as CS+	16	no	Wilcoxon matched-pairs signed rank test	p=0.0250
Figure 6F	R66C08-GAL4 axons training extinction MCH as CS+	11	yes	paired t-test	p=0.2594
Figure S1A	a) 2 pre-exposures 1 min ITI, 10-3	7	na	One sample t test (theoretical mean = 0), ANOVA with Tukey's multiple comparisons test	t-test: p < 0.0001, ANOVA < 0.0001, Turkey's: a vs b < 0.0001, a vs c < 0.0001, b vs c = 0.246
	b) 2 pre-exposures 15 min ITI, 10-3	8	yes		
	c) 15 pre-exposures 1 min ITI, 10-3	8	yes		
	d) 2 pre-exposures 15 min ITI, 10-4	8	yes	One sample t test (theoretical mean = 0)	p = 0.843
	a) 2 pre-exposures 1 min ITI, 10-6	11	yes	One sample t test (theoretical mean = 0)	t-test: a = 0.883, b = 0.290, c = 0.22, ANOVA: p = 0.675
	b) 2 pre-exposures 15 min ITI, 10-6	11	yes		
	c) 5 pre-exposures 1 min ITI, 10-6	11	yes		
Figure S1B	10-3	6	na	paired t-test	p = 0.4852
	10-6	6	na		
Figure S1C left	a) training/extinction/retraining/immediate test	9	yes	ANOVA with Tukey's multiple comparisons test	ANOVA =0.7637
	b) training/---/retraining/immediate test	8	yes		
	c) ---/---/retraining/immediate test	9	yes		
Figure S1C right	a) training/extinction/retraining/ 90 min test	9	yes	ANOVA with Tukey's multiple comparisons test	ANOVA = 0.0137, Turkey's: a vs b = 0.975, a vs c = 0.02, b vs c = 0.03
	b) training/---/retraining/ 90 min test	9	yes		
	c) ---/---/retraining/ 90 min test	9	yes		
Figure S1D	a) R58E02-GAL4 control CS- exposed	6	na	unpaired t-test for comparision between same genotype but different treatment, ANOVA with Tukey's multiple comparisons test for comparision between different genotype, same treatment	ANOVA (CS-) = 0.1770, ANOVA (CS+) = 0.9490, unpaired t-test: a vs b = 0.0003, c vs d = 0.0012, e vs f = 0.02
	b) R58E02-GAL4 control CS+ exposed	6	na		
	c) UAS-Shits1 control CS- exposed	6	na		
	d) UAS-Shits1 control CS+ exposed	6	na		
	e) R58E02-GAL4; UAS-Shits1 CS- exposed	6	na		
	f) R58E02-GAL4; UAS-Shits1 CS+ exposed	6	na		
Figure S1E	a) R58E02-GAL4 CS+ exposed	9	yes	ANOVA with Tukey's multiple comparisons test	ANOVA = 0.411
	c) UAS-shits CS+ exposed	9	yes		
	e) R58E02-GAL4; UAS-Shits1 CS+ exposed	9	yes		
Figure S2A	a) VT1211-GAL4	9	yes	ANOVA with Tukey's multiple comparisons test for comparison	ANOVA = 0.3359
	c) UAS-Shits1	9	yes		
	b) VT1211-GAL4	9	yes		
Figure S2B	a) VT1211-GAL4 control CS- exposed	11	yes	ANOVA with Tukey's multiple comparisons test	ANOVA = 0.5785
	c) UAS-Shits1 control CS- exposed	11	yes		
	b) VT1211-GAL4 control CS- exposed	10	yes		
	d) VT1211-GAL4 control CS+ exposed	11	yes	ANOVA with Tukey's multiple comparisons test for comparison	ANOVA = 0.6085
	e) UAS-Shits1 control CS+ exposed	11	no		
	f) VT1211-GAL4 control CS+ exposed	10	yes		
Figure S3A	R66C08-GAL4 dendrites training no extinction OCT as CS+	11	yes	paired t-test	p=0.9359
Figure S3B	R66C08-GAL4 dendrites training extinction OCT as CS+	13	yes	paired t-test	p=0.0417
Figure S3C	MB112C-GAL4 dendrites training no extinction OCT as CS+	17	yes	paired t-test	p=0.0107
Figure S3D	MB112C-GAL4 dendrites training extinction OCT as CS+	15	no	Wilcoxon matched-pairs signed rank test	p=0.0302
Figure S3E	VT1211-GAL4 dendrites training no extinction MCH as CS+	14	no	Wilcoxon matched-pairs signed rank test	p=0.3258
Figure S3F	VT1211-GAL4 dendrites training extinction MCH as CS+	13	no	Wilcoxon matched-pairs signed rank test	p=0.0215
Figure S3G	VT1211-GAL4 dendrites training no extinction OCT as CS+	14	yes	paired t-test	p=0.3803
Figure S3H	VT1211-GAL4 dendrites training extinction OCT as CS+	11	yes	paired t-test	p=0.0104
Figure S3I	R39A05-GAL4 dendrites training no extinction OCT as CS+	16	yes	paired t-test	p=0.1284
Figure S3J	R39A05-GAL4 dendrites training extinction OCT as CS+	12	yes	paired t-test	p=0.0934
Figure S3K	MB112C-GAL4 dendrites mock no extinction OCT as CS+	11	no	Wilcoxon matched-pairs signed rank test	p>0.999
Figure S3L	MB112C-GAL4 dendrites mock extinction OCT as CS+	12	no	Wilcoxon matched-pairs signed rank test	p=0.7334
Figure S3M	MB112C-GAL4 dendrites mock no extinction MCH as CS+	18	no	Wilcoxon matched-pairs signed rank test	p=0.7987
Figure S3N	MB112C-GAL4 dendrites mock extinction MCH as CS+	15	no	Wilcoxon matched-pairs signed rank test	p=0.9341
Figure S3O	VT1211-GAL4 dendrites mock no extinction MCH as CS+	11	no	Wilcoxon matched-pairs signed rank test	p=0.7002
Figure S3P	VT1211-GAL4 dendrites mock extinction MCH as CS+	11	no	Wilcoxon matched-pairs signed rank test	P=0.8984
Figure S3Q	VT1211-GAL4 dendrites mock no extinction OCT as CS+	11	yes	paired t-test	p=0.8764
Figure 3R	VT1211-GAL4 dendrites mock extinction OCT as CS+	10	no	Wilcoxon matched-pairs signed rank test	p=0.7695
Figure S6A	VT1211-GAL4 axons training no extinction OCT as CS+	11	yes	paired t-test	p=0.0065
Figure S6B	VT1211-GAL4 axons training extinction OCT as CS+	10	yes	paired t-test	p=0.7695
Figure S6C	R66C08-GAL4 axons training no extinction OCT as CS+	24	no	Wilcoxon matched-pairs signed rank test	p=0.0457
Figure S6D	R66C08-GAL4 axons training extinction OCT as CS+	12	no	Wilcoxon matched-pairs signed rank test	p=0.9697
Figure S6E	R39A05-GAL4 axons training no extinction OCT as CS+	14	yes	paired t-test	p=0.0378
Figure S6F	R39A05-GAL4 axons training extinction OCT as CS+	16	no	Wilcoxon matched-pairs signed rank test	p=0.0250
Figure S6G	VT1211-GAL4 axons mock no extinction MCH as CS+	12	no	Wilcoxon matched-pairs signed rank test	p=0.1099
Figure S6H	VT1211-GAL4 axons mock extinction MCH as CS+	11	yes	paired t-test	p=0.2848
Figure S6I	VT1211-GAL4 axons mock no extinction OCT as CS+	10	no	Wilcoxon matched-pairs signed rank test	p=0.7695
Figure S6J	VT1211-GAL4 axons mock no extinction OCT as CS+	11	yes	paired t-test	p=0.1923
